# Transcriptome analysis reveals EBF1 ablation-induced injuries in cardiac system

**DOI:** 10.7150/thno.92060

**Published:** 2024-08-12

**Authors:** Yueheng Wu, Shaoxian Chen, Guiping Huang, Lu Zhang, Liying Zhong, Yi Feng, Pengju Wen, Juli Liu

**Affiliations:** 1 Medical Research Institute, Guangdong Provincial People's Hospital (Guangdong Academy of Medical Sciences), Southern Medical University, Guangzhou, China, 510080.; 2 Guangdong Cardiovascular Institute, Guangdong Provincial People's Hospital (Guangdong Academy of Medical Sciences), Guangzhou, Guangdong, China, 510080.; 3 Guangdong Provincial Key Laboratory of South China Structural Heart Disease, Guangdong Provincial People's Hospital, Guangdong Academy of Medical Sciences, Guangzhou, China, 510080.; 4 Guangdong Provincial Key Laboratory of Clinical Pharmacology, Medical Research Center, Guangdong Provincial People's Hospital (Guangdong Academy of Medical Sciences), Guangzhou, Guangdong, China, 510080.; 5 Guangdong Provincial Key Laboratory of Stem Cell and Regenerative Medicine, Guangdong-Hong Kong Joint Laboratory for Stem Cell and Regenerative Medicine, Guangzhou Institutes of Biomedicine and Health, Chinese Academy of Sciences, Guangzhou, China, 510530.

**Keywords:** transcription factor, cardiac development, cardiomyocyte specification, cardiac remodeling, human pluripotent stem cells

## Abstract

**Rationale:** Regulatory processes of transcription factors (TFs) shape heart development and influence the adult heart's response to stress, contributing to cardiac disorders. Despite their significance, the precise mechanisms underpinning TF-mediated regulation remain elusive. Here, we identify that EBF1, as a TF, is highly expressed in human heart tissues. EBF1 is reported to be associated with human cardiovascular disease, but its roles are unclear in heart. In this study, we investigated EBF1 function in cardiac system.

**Methods:** RNA-seq was utilized to profile EBF1 expression patterns. CRISPR/Cas9 was utilized to knock out EBF1 to investigate its effects. Human pluripotent stem cells (hPSCs) differentiated into cardiac lineages were used to mimic cardiac development. Cardiac function was evaluated on mouse model with Ebf1 knockout by using techniques such as echocardiography. RNA-seq was conducted to analyze transcriptional perturbations. ChIP-seq was employed to elucidate EBF1-bound genes and the underlying regulatory mechanisms.

**Results:** EBF1 was expressed in some human and mouse cardiomyocyte. Knockout of EBF1 inhibited cardiac development. ChIP-seq indicated EBF1's binding on promoters of cardiogenic TFs pivotal to cardiac development, facilitating their transcriptional expression and promoting cardiac development. In mouse, Ebf1 depletion triggered transcriptional perturbations of genes, resulting in cardiac remodeling. Mechanistically, we found that EBF1 directly bound to upstream chromatin regions of cardiac hypertrophy-inducing genes, contributing to cardiac hypertrophy.

**Conclusions:** We uncover the mechanisms underlying EBF1-mediated regulatory processes, shedding light on cardiac development, and the pathogenesis of cardiac remodeling. These findings emphasize EBF1's critical role in orchestrating diverse aspects of cardiac processes and provide a promising therapeutic intervention for cardiomyopathy.

## Background

The potential to generate diverse cardiovascular lineages from human pluripotent stem cells (hPSCs), including human embryonic stem cells (hESC) and human induced pluripotent stem cells (hiPSCs), offers promising avenues for studying human cardiac development and cardiac diseases in controlled environments 1. A comprehensive comprehension of human cardiac development necessitates the identification of functional genes that segregate distinct populations of cardiovascular lineages, particularly cardiomyocytes, using hPSCs platform. Among the various cell types derived from hPSCs, human cardiomyocytes hold significant interest due to their relevance to cardiac diseases such as congenital heart disease and cardiac hypertrophy. Consequently, it becomes imperative to devise strategies that enhance the efficient differentiation of hESCs or hiPSCs into cardiomyocytes. Achieving optimal cardiomyocyte differentiation from hESCs or hiPSCs hinges on deciphering the signaling mechanisms governing the establishment of human cardiomyocytes. This cardiomyocyte specification is tightly regulated through transcriptional reprogramming 2. For example, Mesp1 is the earliest functional transcription factor (TF) of cardiovascular lineages specification 2. The mechanism by which Mesp1 promotes cardiac specification is multi-faceted in which Mesp1 drives dynamic expression of cardiogenic TFs during mesoderm formation 2. The TFs, such as GATA4 and MEF2C, play critical roles in cardiomyocyte specification and differentiation as well 3-5; However, how and when different populations of cardiovascular lineages, like cardiomyocytes, were segregated remain elusive. Unraveling additional functional TFs responsible for human cardiomyocyte specification promises insights into the intricate process of cardiovascular lineage determination.

Cardiac disease is a major cause of morbidity and mortality worldwide 6. After exposure to pathological signaling, such as sustained pathological mechanical pressure, the myocardium undergoes pathological remodeling, often coupled with cardiomyopathy on the late stage, such as cardiac hypertrophy 7. Under the chronic stimulation of pathological signaling, cardiac hypertrophy results from the reactivation of fetal genes 8. This process is driven by TF-mediated transcriptional reprogramming, guiding the spatiotemporal expression patterns of hypertrophic genes 9. Intriguingly, the TFs, such as GATA4 and MEF2, pivotal in cardiac development and cardiomyocyte specification 3-5, also participate in inducing cardiac hypertrophy 10, 11. This suggests a potential overlap in the transcriptional regulatory networks governing cardiac development and hypertrophy. Hence, exploring TF functionality during human cardiac development provides an alternative avenue for comprehending human cardiac hypertrophy. Elucidating TF roles in human cardiac development enriches our understanding of both heart development and the progression of cardiac diseases.

To elucidate TFs governing human cardiac development, we conducted RNA-seq analyses, comparing differentially expressed genes in right ventricle (RV), left ventricle (LV), sinoatrial node (SN) from health heart tissue with those in human induced pluripotent stem cells (hiPSCs). We hypothesized that candidate TFs specifically expressed in the human heart tissues might underlie cardiac development. Our RNA-seq results highlighted the transcription factor EBF1 (Early B Cell Factor 1), previously unexplored in the context of heart development. EBF1 has been implicated in regulating epigenetic and transcriptional events in B-cell programming and development 12-14, leukemogenesis 15, cancer 16 and cell lineages formation 17, 18. Recent systems biology and genome-wide association studies link EBF1 to various human cardiovascular diseases, such as coronary heart disease 19-22, cardiovascular metabolic disease 23 and orthostatic hypotension 24, implying its potential roles in the human cardiac system. However, EBF1's functions in human heart development and cardiac diseases remain unknown. This study leveraged an iPSCs model to investigate EBF1's role in human cardiac development. The *in vivo* system of mouse model demonstrated that EBF1 depletion induces transcriptional perturbations in heart, resulting in cardiac remodeling, including hypertrophy and fibrosis. These discoveries illuminate EBF1's role in connecting cardiac development and remodeling in human and mouse, thereby enhancing our understanding of transcriptional regulation in cardiac system. Additionally, this research identifies EBF1 as a potential therapeutic target for managing cardiac remodeling.

## Results

### RNA-seq reveals the expression patterns of EBF1 in human heart tissues and cardiac development

**Figure 1:**
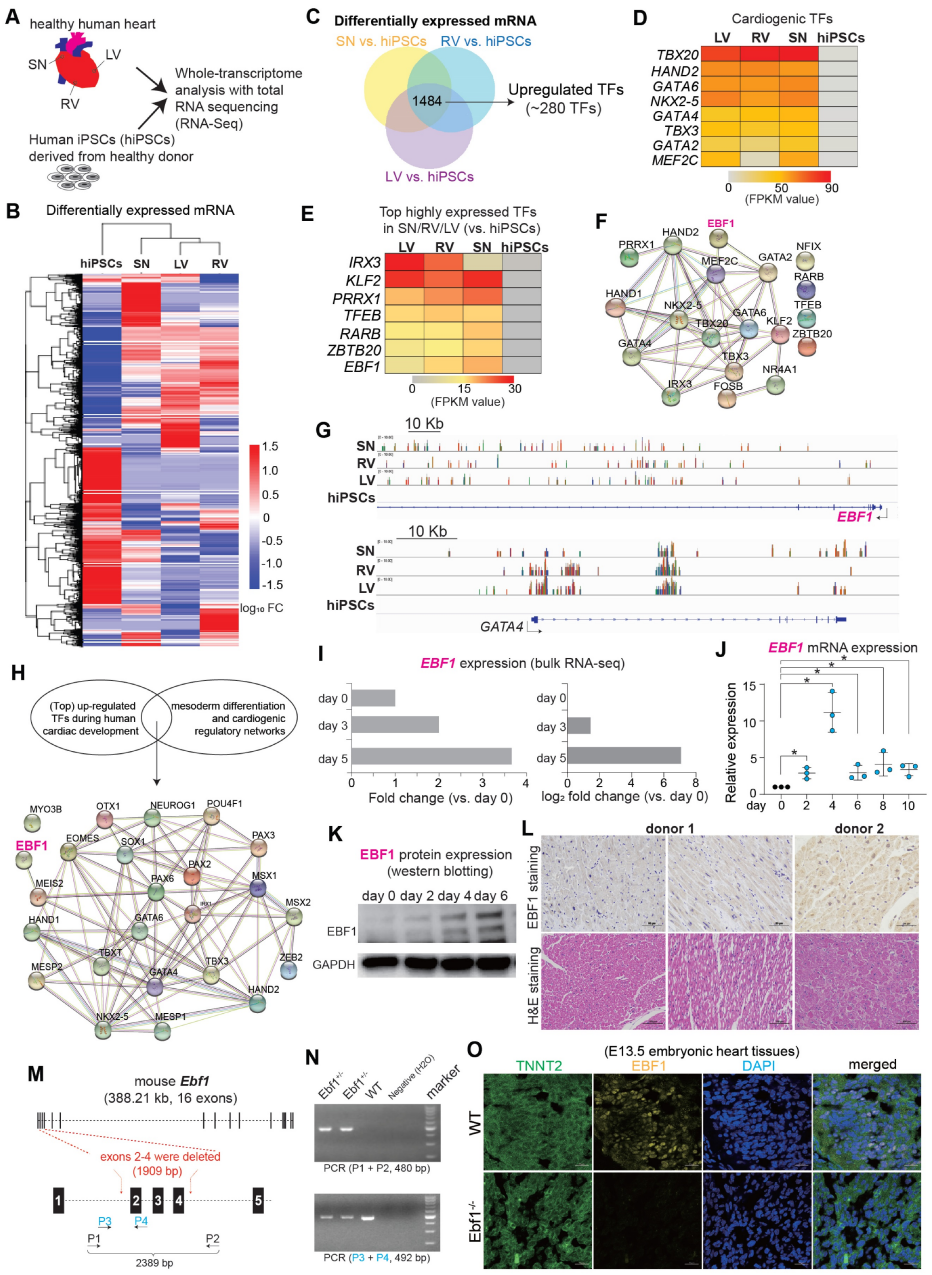
** RNA-seq reveals the expression patterns of EBF1 in human heart tissues and cardiac development.** (A) RNA-seq analysis of healthy human heart tissues (LV, RV, SN) and hiPSCs. RV, human right ventricle tissue; LV, human left ventricle tissue; SN, human sinoatrial node tissue. hiPSCs, human induced pluripotent stem cells. (B) Heat map showing differentially expressed mRNA genes (DEGs). FC, fold change. (C) DEGs analysis showing up-regulated transcription factors (TFs) in LV/RV/SN (vs. hiPSCs). (D) Heat map showing expression levels of TFs, which are crucial for cardiogenesis. (E) Heat map showing expression levels of other top-ranked TFs. (F) Protein-protein interaction (PPI) analysis showing protein-protein interaction networks in cardiac system. PPI was run on STRING (https://string-db.org/). (G) Representative RNA-seq peak tracks. Black arrows showing the transcriptional direction of *EBF1* and *GATA4*. Scale was [0-10.00]. (H) PPI showing protein-protein interaction networks in cardiac system. PPI was run on STRING (https://string-db.org/). (I) RNA-seq data showing *EBF1* expression pattern in hiPSCs (day 0), mesodermal cells (cardiac differentiation on day 3) and cardiac progenitor cells (cardiac differentiation on day 5). (J) RT-qPCR showing *EBF1* dynamic expression changes during human cardiac development of hESCs. *p<0.05. (K) Western-blotting showing EBF1 protein expression pattern during human cardiac development of hESCs. (L) Histochemistry showing the protein expression of EBF1 in healthy human heart tissues. (M) Mouse model with *Ebf1* knockout. Exons 2-4 were deleted by CRISPR/Cas9. P, PCR primer. (N) PCR validation showing the Ebf1 knockout in mouse model. (O) Immunostaining showing EBF1 protein expression in mouse embryonic heart tissues at E13.5 stage. Scale bar, 20 µm.

To understand gene expression profiles in human cardiac development, we performed RNA-seq on health human heart tissues of right ventricle (RV), left ventricle (LV), sinoatrial node (SN) and human induced pluripotent stem cells (hiPSCs) (Figure 1A-B, Figure S1A-F). We hypothesized that TF candidates, exhibiting specific and robust expression in human heart tissues (Figure 1C), would likely participate in human cardiac development, since many cardiogenic TFs (inducers), which have been reported to govern cardiac development, were significantly upregulated in LV/RV/SN 25-28 (Figure 1D). Furthermore, we found highly expressed TFs in LV/RV/SN, such as IRX3, KLF2, PRRX1, TFEB, RARB and ZBTB20 (Figure 1E), all of which have established roles in heart function 29-35. Conversely, the functional implications of EBF1, which ranked prominently among the highly expressed genes (Figure 1E-G), remained unexplored within the context of human cardiac biology. The protein-protein interaction (PPI) analyses suggested that EBF1 might potentially interface with regulatory networks associated with cardiac development (Figure 1F), which indicated its potential role in human cardiac system.

To know EBF1 expression pattern during human cardiac development, we established an *in vitro* model mimicking human cardiac development by differentiating hiPSCs into cardiac lineages, such as mesodermal cells, cardiac progenitor cells and cardiomyocytes (Figure S1G) 36. Within this model, genes pertinent to heart development and cardiac chamber morphogenesis were upregulated, including critical TFs governing mesoderm formation and cardiogenesis, as evidenced by significant upregulation during differentiation (Figure S1H-I). The PPI analysis unveiled potential interactions between EBF1 and the regulatory networks of cardiogenesis (Figure 1H). Notably, *Ebf1* displayed dynamic upregulation throughout the *in vitro* differentiation of human cardiomyocytes from hiPSCs (Figure 1I), a trend consistent with our earlier findings (Figure 1J-K, Figure S1J). Additionally, we also found that EBF1 protein was expressed in intact adult human heart tissues (Figure 1L) and hESC-derived cardiomyocytes (Figure S1K). In order to evaluate the expression of EBF1 protein in different mouse cardiac cell types, we generate wild-type (WT) and *Ebf1* knockout (*Ebf1*^+/-^) mouse model (Figure 1M-N). In embryonic stage E13.5 mouse tissues, EBF1 protein was expressed in WT cardiomyocytes, but its expression was significantly reduced in *Ebf1*^+/-^ cardiomyocytes (Figure 1O). Additionally, EBF1 protein was expressed in cardiomyocytes and cardiac endothelial cells, with much lower levels observed in cardiac smooth muscle cells (Figure S1L).

In summary, our findings established that EBF1, which is expressed in cardiac cells, undergoes upregulation during human cardiac development. This discovery, coupled with the association between EBF1 and cardiovascular diseases highlighted by genome-wide association studies (GWAS) 19-22, led us to speculate that EBF1 can play a role in human cardiac development and the specification of cardiomyocytes.

### EBF1 depletion inhibits human cardiac development

**Figure 2:**
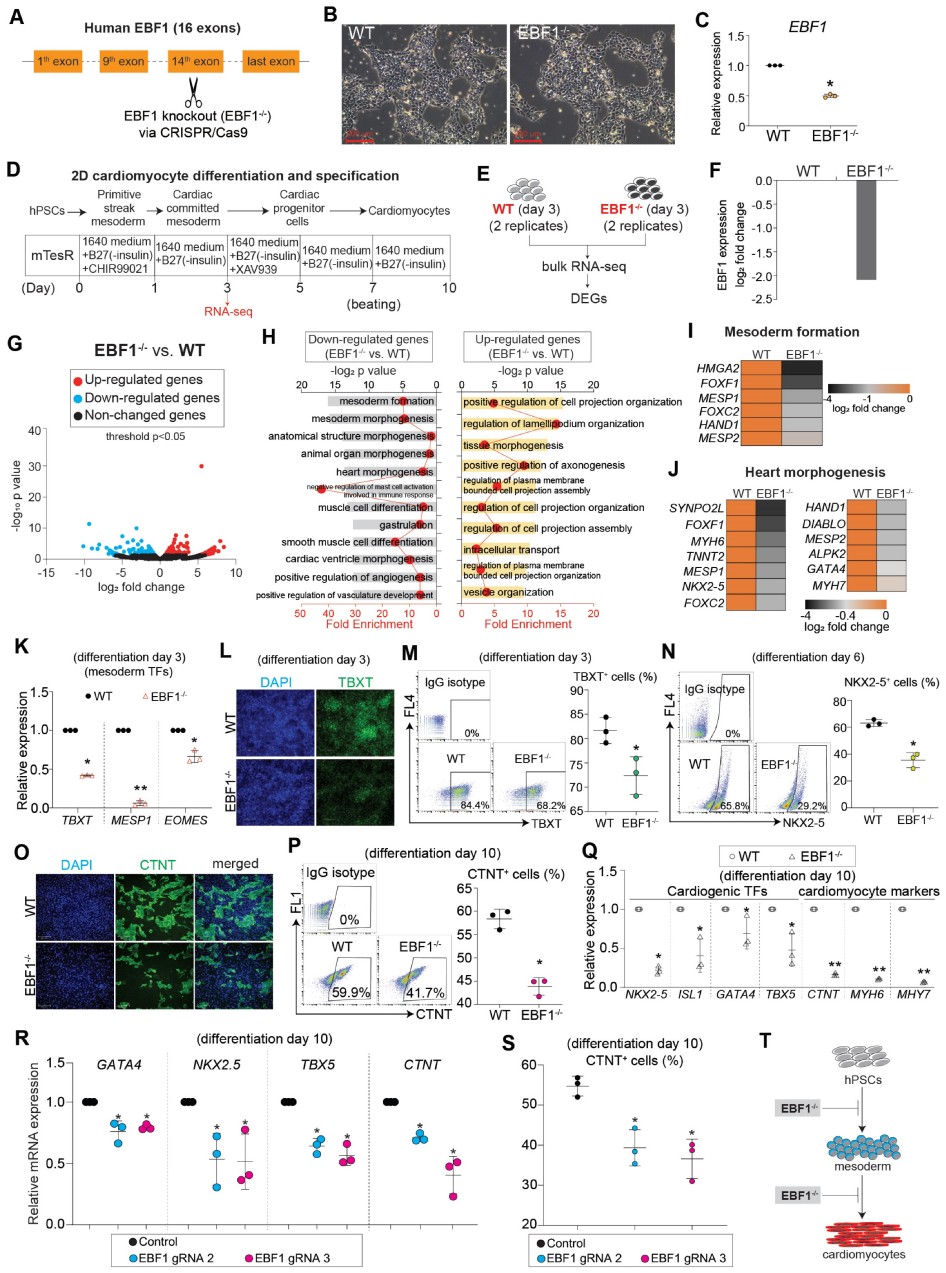
** RNA-seq reveals that EBF1 depletion inhibits human cardiac development.** (A) EBF1 was knocked out (EBF1^-/-^) in H9 human embryonic stem cells (hESCs) by utilizing CRISPR/Cas9. (B) WT and EBF1^-/-^ hESCs maintained in mTesR1 medium. Scale bar, 200 µm. (C) RT-QPCR showing *EBF1* expression level in WT and EBF1^-/-^ hESCs. *p<0.05 (EBF1^-/-^ vs. WT). (D-E) 2D model of human cardiac development to study EBF1 function. Both WT and EBF1^-/-^ cells on day 3 post differentiation were collected for RNA-seq, followed with analyses of differentially expressed genes (DEGs). Two biological replicates were performed for RNA-seq. (F) RNA-seq showing relative expression level of *EBF1* between WT and EBF1^-/-^ cells (EBF1^-/-^ vs. WT). (G) Volcano plot showing gene expression profile between WT and EBF1^-/-^ cells (EBF1^-/-^ vs. WT). (H) Gene Ontology (GO) analysis showing top GO terms for differentially expressed genes (EBF1^-/-^ vs. WT). (I-J) Heat map showing relative expression levels of genes important for mesoderm formation (I) and heart morphogenesis (J). (K) RT-QPCR showing expression levels of mesodermal TFs (*TBXT, MESP1, EOMES*) (cardiac differentiation on day 3). *p<0.05 (EBF1^-/-^ vs. WT); **p<0.001 (EBF1^-/-^ vs. WT). (L) Immunostaining showing TBXT protein expression in WT and EBF1^-/-^ cells (cardiac differentiation on day 3). Green was TBXT staining. Blue was DAPI (nuclei) staining. Scale bar, 100 µm. (M) Flow cytometry showing percentage (%) of TBXT positive (TBXT^+^) cells (cardiac differentiation on day 3). *p<0.05 (EBF1^-/-^ vs. WT). (N) Flow cytometry showing percentage (%) of NKX2-5 positive (NKX2-5^+^) cells (cardiac differentiation on day 6). *p<0.05 (EBF1^-/-^ vs. WT). (O) Immunostaining showing CTNT positive (CTNT^+^) cells (cardiac differentiation on day 10). CTNT is one of the specific sarcomere markers controlling cardiomyocyte contraction. Scale bar, 200 µm. (P) Flow cytometry showing percentage (%) of CTNT positive (CTNT^+^) cells (cardiac differentiation on day 10). *p<0.05 (EBF1^-/-^ vs. WT). (Q) RT-QPCR showing mRNA expression levels of cardiogenic TFs (*NKX2-5, ISL1, GATA4, TBX5*) and specific cardiomyocyte markers (*CTNT, MYH6, MYH7*) (day 10). *p<0.05 (EBF1^-/-^ vs. WT); **p<0.001 (EBF1^-/-^ vs. WT). (R) RT-QPCR showing mRNA expression levels of cardiogenic TFs (*NKX2-5, GATA4, TBX5*) and specific cardiomyocyte marker (*CTNT*) (differentiation on day 10). *p<0.05 (EBF1 gRNA2 vs. Control or EBF1 gRNA3 vs. Control). (S) Flow cytometry showing percentage (%) of CTNT positive (CTNT^+^) cells (day 10). *p<0.05 (EBF1 gRNA2 vs. Control or EBF1 gRNA3 vs. Control). (T) The function of EBF1 in human cardiac development. EBF1 depletion inhibits human cardiac development. hPSCs, human pluripotent stem cells.

To interrogate the role of EBF1, we employed a knockout strategy to eliminate EBF1 in human embryonic stem cells (hESCs) (Figure 2A-C, Figure S2A). Subsequently, we induced the differentiation of these hESCs into cardiomyocytes using a 2D differentiation system (Figure 2D), a methodology built upon our previous works, which can mimic human cardiac development 36-38. To elucidate the function of EBF1, we collected both wild-type (WT) and *EBF1* knockout (EBF1^-/-^) cells on day 3 post differentiation for RNA-seq analysis, allowing us to identify differentially expressed genes (DEGs) (Figure 2D-E). RNA-seq analysis exhibited reduced *EBF1* in EBF1^-/-^ cells compared to WT cells (Figure 2F), resulting in transcriptional perturbation (Figure 2G). Further enrichment analysis using gene ontology (GO) revealed that EBF1 potentially played a critical role in inhibiting mesoderm formation (Figure 2H-I) and impeding heart morphogenesis (Figure 2H-J). These findings demonstrated the potential importance of EBF1 in human mesoderm and cardiogenesis. To validate that, we examined the expression of representative markers for cardiac lineages, including mesodermal cell markers (*TBXT, MESP1, EOMES*), cardiac progenitor cell marker (*NKX2-5*), and cardiomyocyte marker (*TNNT2*). This evaluation aimed to assess whether depleting EBF1 would impact cardiac development. Our findings demonstrated that EBF1 knockout significantly repressed the expression of mesoderm markers (Figure 2K) and reduced the percentage of TBXT^+^ cells (Figure 2L-M). Moreover, EBF1 depletion resulted in a decrease in the percentage of NKX2-5^+^ (Figure 2N) and CTNT^+^ (Figure 2O-P) cells. Additionally, cardiogenic TFs (*NKX2-5, ISL1, GATA4* and *TBX5*) and cardiomyocyte contraction markers (*CTNT, MYH6, MYH7*) were all down-regulated by EBF1 knockout (Figure 2Q). To further confirm the role of EBF1, we utilized two new gRNAs that effectively knocked out EBF1 (Figure S2A-B). Notably, these gRNAs caused down-regulation of mesodermal and cardiogenic markers (Figure 2R, Figure S2C) and finally decreased the percentage of CTNT^+^ cells (Figure 2S). In summary, our results provided compelling evidence that EBF1 depletion inhibited human cardiac development, affecting both mesoderm differentiation and cardiomyocyte specification (Figure 2T).

### EBF1 is required for proper expression of mesodermal and cardiogenic TFs via its binding on promoters

**Figure 3:**
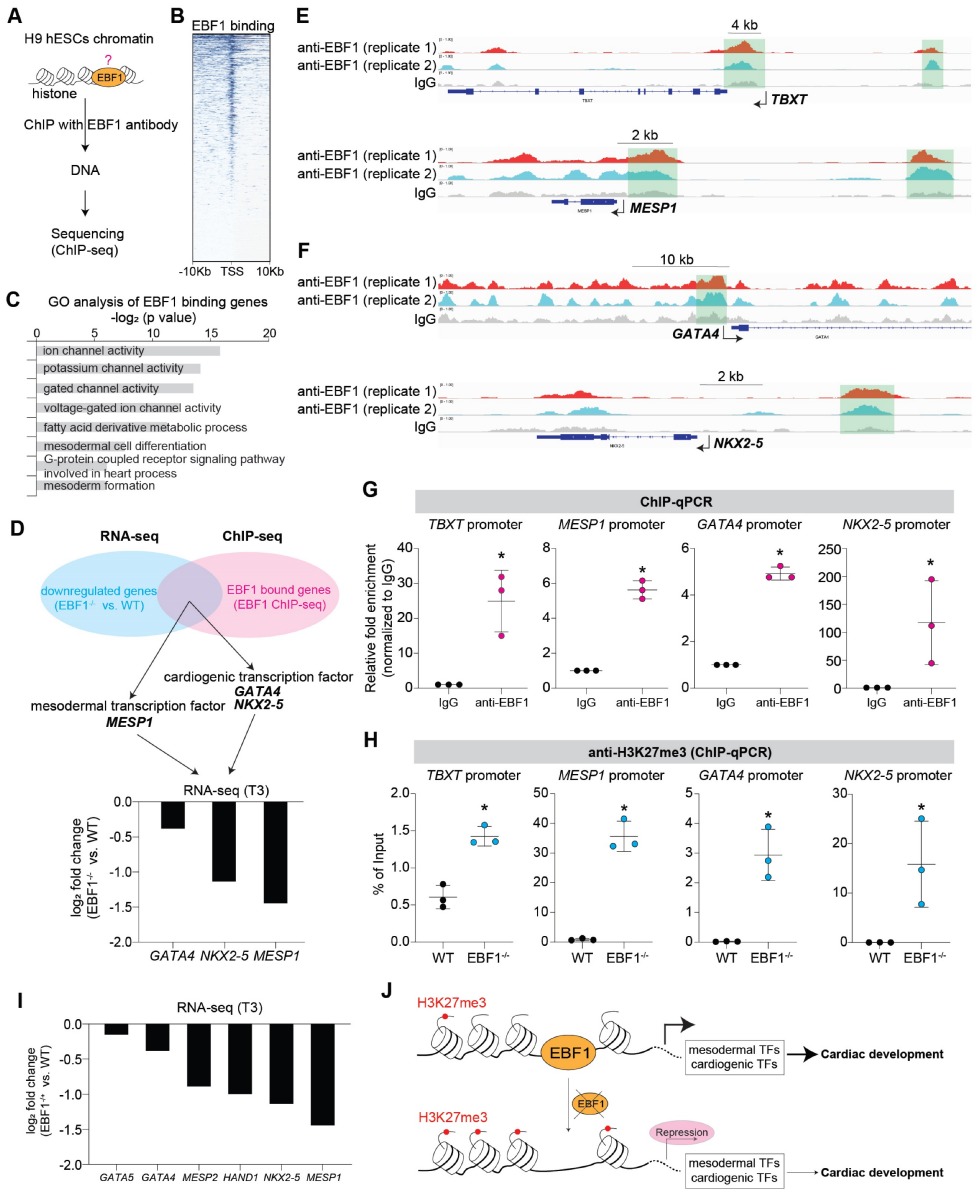
** EBF1 is required for proper expression of mesodermal and cardiogenic TFs via its binding on promoters.** (A) Chromatin immunoprecipitation followed with sequencing (ChIP-seq) to evaluate EBF1 binding on chromatin in H9 hESCs. Two biological replicates were performed for ChIP-seq. (B) Intensity plots showing the read densities over the peaks (± 10 kb) in profiles. TSS, near transcriptional start sites. (C) Gene Ontology (GO) analysis for EBF1 binding genes. GO was run on GENEONTOLOGY. GO terms (the biological process) with p<0.05 were selected. (D) Overlapped genes of RNA-seq and ChIP-seq data. RNA-seq was from Figure 2E. (E) Representative ChIP-seq peaks of EBF1 binding on mesodermal inducers (TFs) (*TBXT, MESP1*). Highlighted green boxes showing EBF1 binding regions. Anti-EBF1, antibody specifically targeting EBF1; IgG, isotype antibody for negative control. Scale was [0-1.00]. (F) Representative ChIP-seq peaks of EBF1 binding on cardiogenic inducer (TFs) (*GATA4, NKX2-5*). Highlighted green dotted boxes showing EBF1 binding regions. Anti-EBF1, antibody specifically targeting EBF1; IgG, isotype antibody as the negative control. Scale was [0-1.00]. (G) ChIP-qPCR showing EBF1 binding on promoters of *TBXT, MESP1, GATA4* and *NKX2-5* in hESCs. *p<0.05 (anti-EBF1 vs. IgG). (H) ChIP-qPCR showing H3K27me3 binding on promoters of *TBXT, MESP1, GATA4* and *NKX2-5* in WT and EBF1^-/-^ hESCs. *p<0.05 (EBF1^-/-^ vs. WT). (I) RNA-seq showing relative expression levels of mesodermal and cardiogenic TFs. T3, cardiac differentiation on day 3. (J) The working model showing the EBF1 regulatory mechanism controlling human cardiac development.

To investigate the mechanism of EBF1 as a transcription factor in human cardiac development, we conducted chromatin immunoprecipitation followed by sequencing (ChIP-seq) to investigate EBF1's genomic binding profile (Figure 3A). ChIP-seq results unveiled EBF1's preferential binding to chromatin DNAs in proximity to transcriptional start sites (TSS) (Figure 3B). Gene Ontology (GO) analyses shed light on the functional significance of EBF1-bound genes, revealing their involvement in processes related to mesoderm differentiation and heart development (Figure 3C).

We further overlapped RNA-seq and ChIP-seq results and found that *MESP1, GATA4* and *NKX2-5* were in the overlapped genes (Figure 3D-F). Although *TBXT* was not one of the overlapped genes, ChIP-seq revealed that EBF1 could also bind on *TBXT* promoter (Figure 3E). This indicated that these genes were EBF1 downstream targets. MESP1 is expressed in mesoderm and a pivotal transcription factor (TF) during cardiac fate determination and drives cardiac differentiation 39-46. MESP1 knockout or repression can lead to repression in cardiac differentiation and cardiomyocyte specification 39-41, 44, 45. TBXT, also named as BRACHYURY, is the key mesoderm specifier and TF. It is critical for mesoderm induction, heart development and cardiac differentiation 37, 47-52. The transcription factors GATA4 and NKX2-5 are critical regulators of cardiac gene expression and can drive cardiac differentiation and cardiomyocyte formation 53-58. These evidences suggested EBF1's potential roles in mesoderm differentiation and cardiomyocyte specification. ChIP-qPCR further confirmed that EBF1 could occupy these promoters (Figure 3G). Furthermore, we found that EBF1 ablation led to increased binding of H3K27me3 on promoters of these TFs (Figure 3H). H3K27me3 is a marker showing repressive transcription 59. Consequently, EBF1 ablation-induced increased binding of H3K27me3 on promoters contributed to the expression repression of these cardiogenic TFs (Figure 2, Figure 3I), which finally inhibited cardiac development (Figure 2).

These findings collectively corroborated the evidence of EBF1 role in repressing mesoderm and cardiomyocyte formation upon its depletion. Mechanistically, EBF1 bound to these gene promoters, facilitating the subsequent activation of transcriptional expression for mesodermal and cardiogenic TFs. Taken together, our study demonstrated that EBF1 emerged as a promoter of human cardiac development through its ability to bind to the promoters of mesodermal and cardiogenic TFs, thereby activating their transcriptional expression (Figure 3J).

### EBF1 depletion affects lineage specification and induces DNA damage

**Figure 4:**
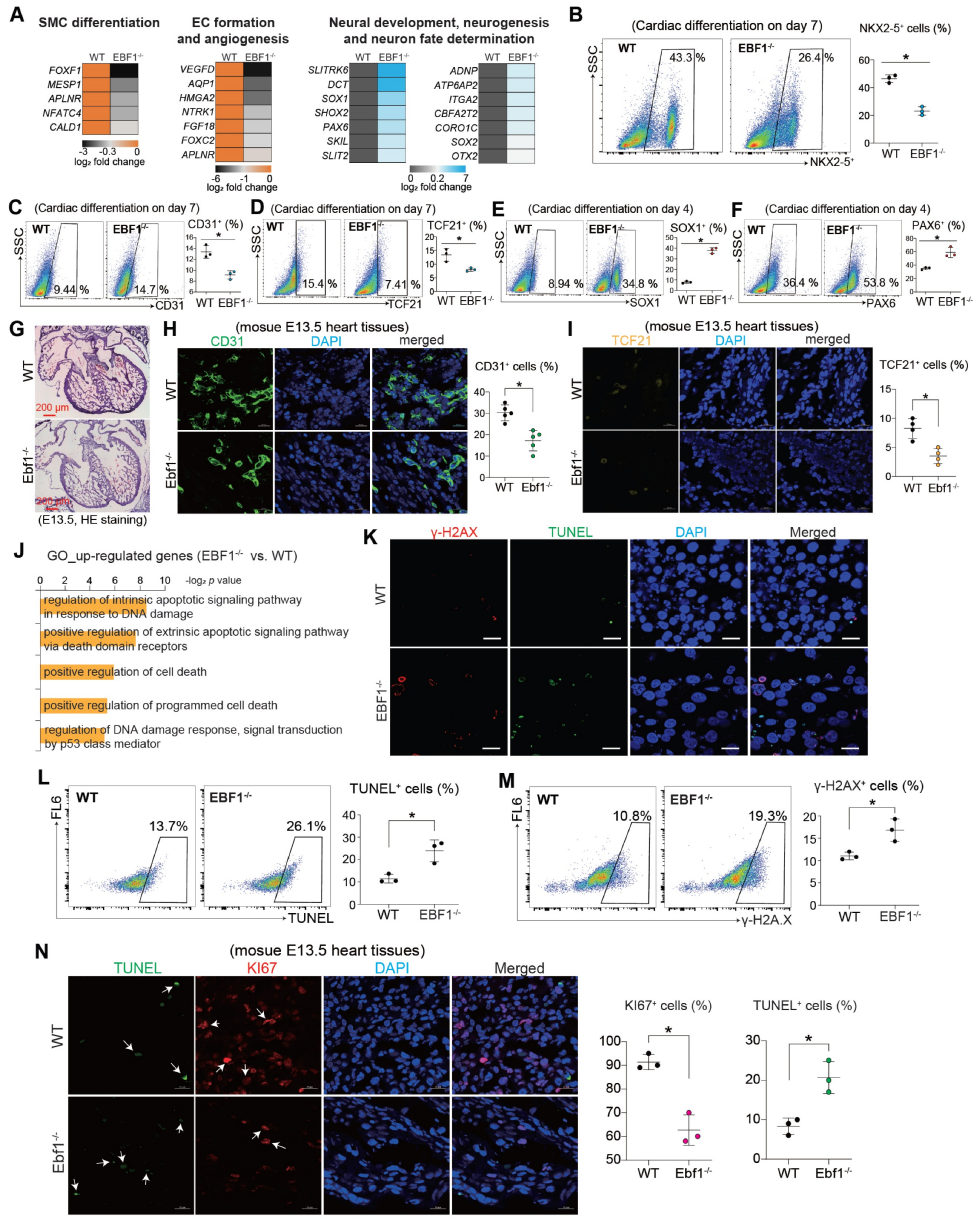
** EBF1 depletion affects lineage specification and induces DNA damage.** (A) Heat map showing relative expression levels of genes important for smooth muscle cell (SMC) differentiation, endothelial cell (EC) formation and neural development. (B) Flow cytometry showing the percentage (%) of NKX2-5^+^ cells on day 7 post cardiac differentiation. *p<0.05. (C-D) Flow cytometry showing the percentage (%) of CD31^+^ (C) and TCF21^+^ (D) cells on day 7 post cardiac differentiation. *p<0.05. (E-F) Flow cytometry showing the percentage (%) of SOX1^+^ (E) and PAX6^+^ (F) cells on day 4 post cardiac differentiation. *p<0.05. (G) Hematoxylin and eosin (HE) staining of mouse E13.5 heart tissues (embryonic E13.5 stage). Scale bar, 200 µm. (H) Immunostaining showing CD31 expression in mouse E13.5 heart tissues. CD31 is one of the specific endothelial cells (EC) markers. Scale bar, 20 µm. *p<0.05. (I) Immunostaining showing TCF21 expression in mouse E13.5 heart tissues. TCF21 is one of the specific smooth muscle cells (SMC) markers. Scale bar, 20 µm. *p<0.05. (J) Gene Ontology (GO) analysis showing cell death signaling induced by EBF1 depletion. (K) Immunostaining to evaluate the DNA damage by detecting γ-H2AX (an early cellular response to DNA double-strand breaks) and TUNEL (a marker for DNA damage and cell death) in cells on day 3 post differentiation. Scale bar, 50 µm. (L-M) Flow cytometry quantifying the percentages of TUNEL^+^ (L) and γ-H2AX^+^ (M) cells on day 3 post differentiation. *p<0.05 (EBF1^-/-^ vs. WT). (N) Immunostaining to evaluate the expression of TUNEL (a marker for DNA damage and cell death) and KI67 (a proliferation marker) in mouse E13.5 heart tissues. Scale bar, 50 µm. *p<0.05.

Whether the repression of cardiac development by EBF1^-/-^ could extend to other lineages differentiation remained unexplored. Intriguingly, RNA-seq analysis revealed that EBF1 knockout led to reduced expression levels of genes crucial for the formation of smooth muscle cells (SMC) and endothelial cells (EC) (Figure 4A), whereas expression levels of neural markers were increased by EBF1 knockout (Figure 4A). Consequently, we therefore evaluated the expression of SMC marker (α-SMA), EC marker (CD31) and fibroblasts (FB) marker (TCF21). EBF1 depletion not only decreased the percentage of NKX2-5^+^ cells (a cardiomyocyte marker, Figure 4B), but also decreased the percentage of α-SMA^+^ cells (Figure S2D), CD31^+^ cells (Figure 4C) and TCF21^+^ cells (Figure 4D). These findings demonstrated that EBF1 depletion represses the specifications of cardiac lineages. Conversely, EBF1 knockout resulted in increased percentage of SOX1^+^ cells (Figure 4E) and PAX6^+^ cells (Figure 4F), suggesting that neural differentiation is promoted following EBF1 depletion. Additionally, EBF1 knockout decreased the percentage of CD31^+^ cells (Figure 4G-H) and TCF21^+^ cells (Figure 4I) in mouse heart tissues at the E13.5 stage. These findings demonstrate that EBF1 depletion affects lineage specification both* in vitro* and *in vivo*.

We noted that EBF1^-/-^-upregulated genes were linked to DNA damage and cell death (Figure 4J). Therefore, we next evaluated the DNA damage by detecting γ-H2AX (an early cellular response to DNA double-strand breaks) and TUNEL (a marker for DNA damage and cell death) in cardiac development using a human pluripotent stem cell (hPSC) model. Our results showed that EBF1^-/-^ cells had a higher percentage of γ-H2AX^+^ and TUNEL^+^ cells compared to WT cells (Figure 4K-M). Similarly, in E13.5 mouse heart tissues, EBF1 knockout increased the percentage of TUNEL^+^ cells and decreased KI67^+^ cells (Figure 4N). These results indicate that EBF1 depletion induces DNA damage.

These findings collectively underscored the influence of EBF1 depletion on the differentiation of diverse lineages and its potential role in inducing DNA damage.

### EBF1 depletion induces transcriptional perturbations in adult mouse heart tissues

**Figure 5:**
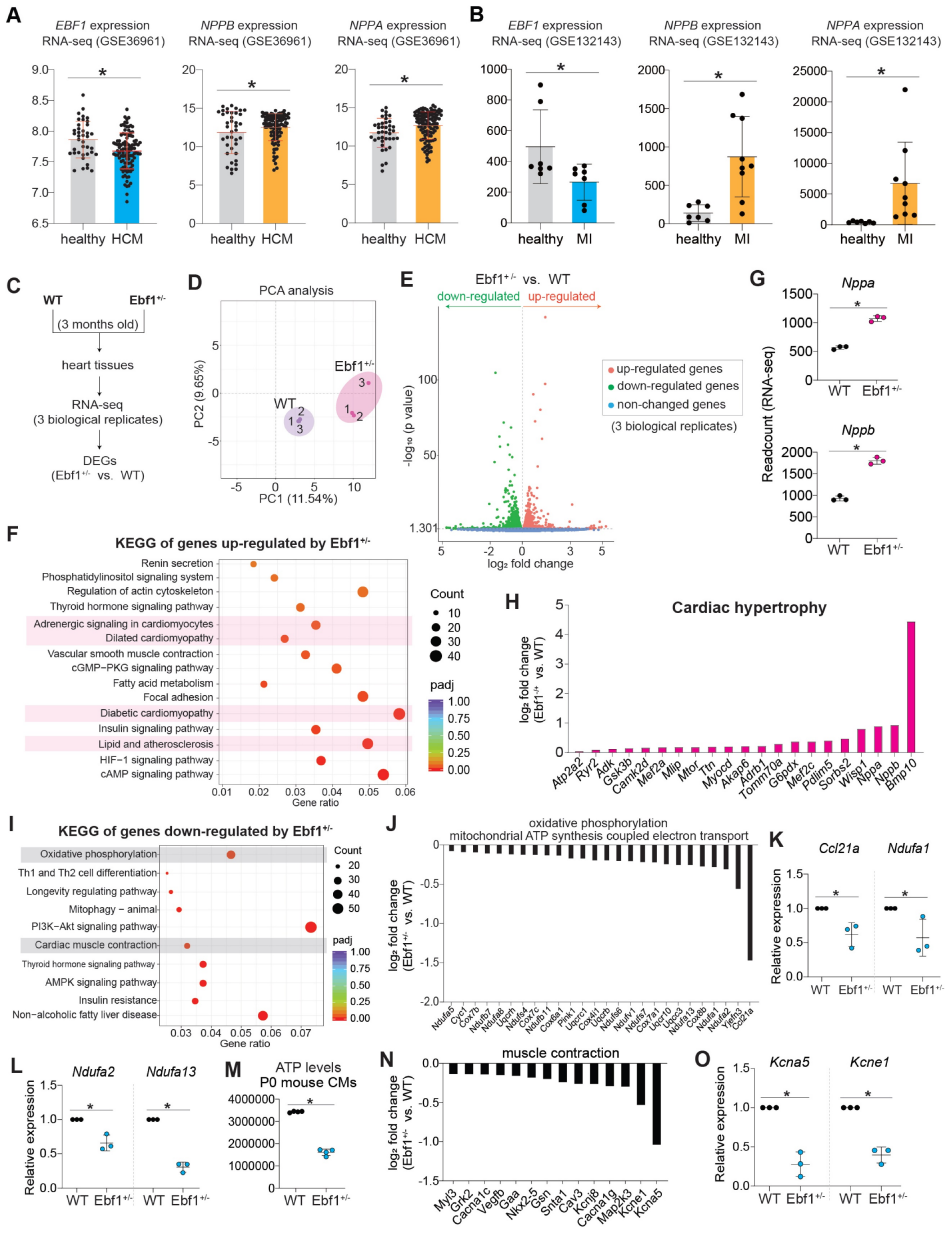
** EBF1 deficiency induces transcriptional perturbations in adult mouse heart.** (A-B) RNA-seq showing *EBF1* expression in human heart tissues. HCM, hypertrophic cardiomyopathy. MI, myocardial infraction. In (A), Y-axis is the RNA-seq read counts. *p <0.05. In (B), Y-axis is the RNA-seq FPKM values. *p <0.05. *NPPA* and *NPPB* are indicative markers of cardiac remodeling, including cardiac hypertrophy and fibrosis. (C-E) RNA-seq of 3-month-old mouse heart tissues. DEGs, differentially expressed genes. PCA, Principal Component Analysis. (F) KEGG analysis of up-regulated genes induced by *Ebf1*^-/-^ in mouse heart tissues. (G-H) The expression levels of genes associated with cardiac hypertrophy. *p <0.05. (I) KEGG analysis of down-regulated genes induced by *Ebf1*^-/-^ in mouse heart tissues. (J) RNA-seq showing the relative expression levels of genes associated with ATP biosynthesis in mouse heart tissues. (K-L) RT-QPCR showing the relative expression levels of the critical and essential genes controlling ATP biosynthesis in mouse heart tissues. *p <0.05. (M) Cellular ATP levels evaluation in cardiomyocytes from neonatal mouse heart (P0 mouse CMs). *p <0.05. (N) RNA-seq showing the relative expression of genes controlling muscle contraction. (O) RT-qPCR showing expression levels of genes controlling cardiac conduction. *p <0.05.

We discovered that EBF1 depletion caused DNA damage (Figure 4J-N). EBF1 is expressed in both human and mouse heart tissues (Figure 1, Figure S3A-B). Additionally, we found that EBF1 was significantly downregulated in hypertrophic cardiomyopathy (HCM) heart tissues (Figure 5A) and myocardial infraction (MI) heart tissues (Figure 5B), compared to healthy heart tissues, respectively. These data indicated a potential involvement of EBF1 in cardiac injuries. To further investigate its function, we next evaluated whether Ebf1 knockout could affect cardiac function in adult mice.

To globally understand the role of *Ebf1* in heart, we conducted RNA-seq on WT and *Ebf1*^+/-^ adult mouse heart tissues (Figure 5C-D). The transcriptomic analysis unveiled significant transcriptional perturbations induced by *Ebf1*^+/-^ (Figure 5E), with up-regulated genes linked to cardiomyopathy (Figure 5F). Two specific cardiac remodeling markers, *Nppa* and *Nppb*, were significantly up-regulated in *Ebf1*^+/-^ compared to WT (Figure 5G). Moreover, the expressions of other cardiac remodeling-associated markers were also up-regulated in *Ebf1*^+/-^ compared to WT (Figure 5H). Thus, this evidence demonstrated that EBF1 depletion instigates cardiac hypertrophy, which are similar with some phenotypes reported in the public database (informatics.jax.org/marker/MGI:95275) (Figure S3C-D).

KEGG analysis of RNA-seq data revealed that *Ebf1*^+/-^-downregulated genes were linked to oxidative phosphorylation/ATP biosynthesis and cardiac muscle contraction (Figure 5I-J). RT-QPCR confirmed that the critical genes controlling ATP synthesis were significantly downregulated in *Ebf1*^+/-^ compared to WT (Figure 5K-L), leading to decreased ATP generation (Figure 5M). RT-QPCR also showed that genes essential for muscle contraction were repressed by EBF1 knockout in heart tissues (Figure 5N-O).

Thus, our findings demonstrated that EBF1 depletion induces transcriptional perturbations in adult mouse heart, which may lead to cardiomyopathy.

### EBF1 depletion induces cardiac remodeling in adult mouse

**Figure 6:**
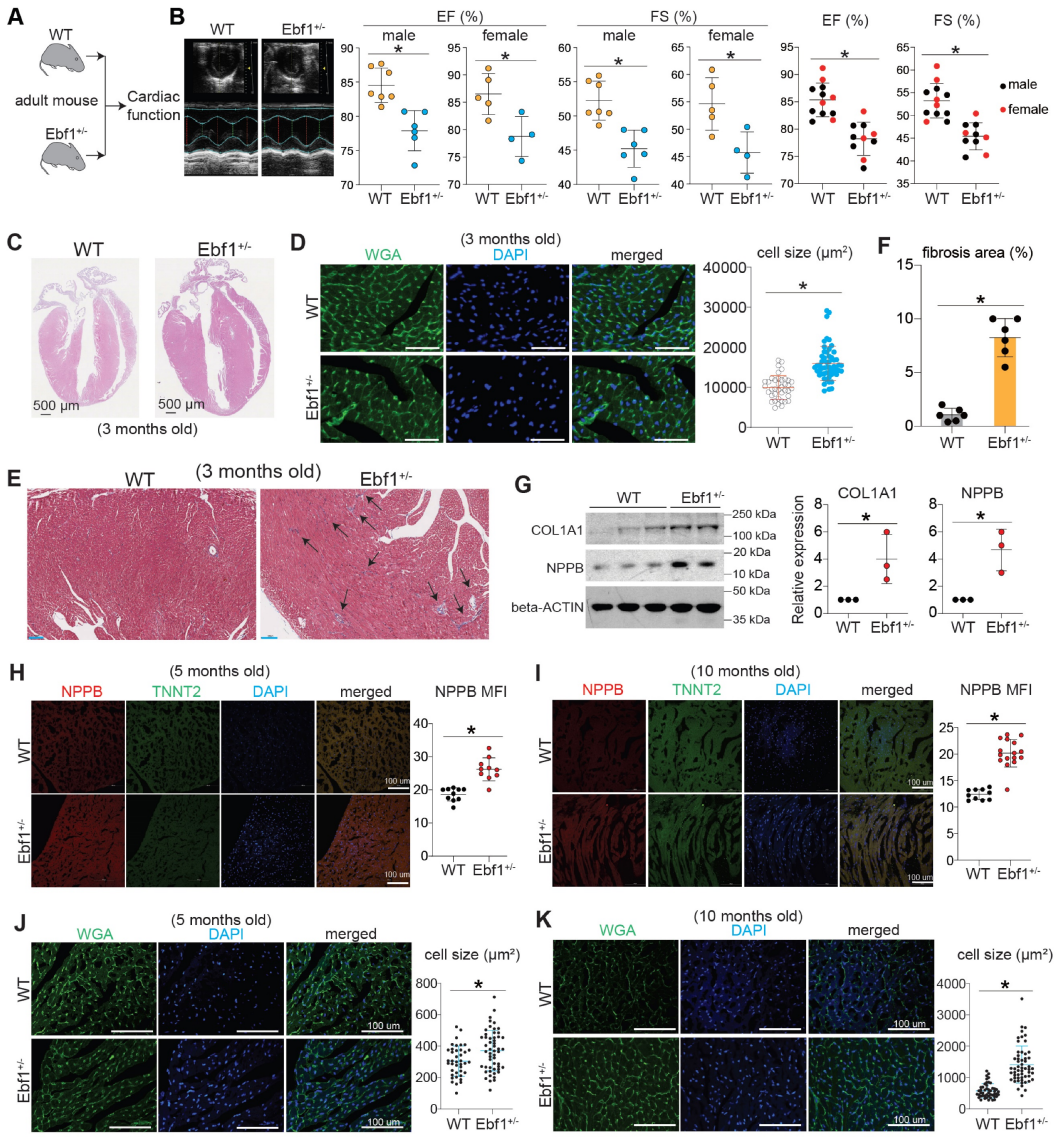
** EBF1 depletion induces cardiac remodeling in adult mouse.** (A-B) Echocardiography showing the ejection fraction (EF, %) and fractional shortening (FS, %) of 3-month-old mice. Echocardiography was performed on conscious mouse. *p <0.05 (*Ebf1*^+/-^ vs. WT). (C) Hematoxylin and eosin (HE) staining on mouse heart sections of 3-month-old mice. Scale bar, 500 µm. (D) WGA staining on mouse heart sections of 3-month-old mice. Cardiomyocyte size was quantified. *p <0.05. Scale bar, 200 µm. (E-F) Masson staining on mouse heart sections of 3-month-old mice. Arrows showed the staining of extracellular matrix (fibrosis) (E). The percentage of fibrosis area was quantified (F). Scale bar, 100 µm. (G) Western blot showing the protein expression of cardiac hypertrophy marker (NPPB) and cardiac fibrosis marker (COL1A1). *p <0.05. (H) Immunostaining of NPPB on mouse heart sections of 5-month-old mice. The MFI (mean fluorescence intensity), showing NPPB expression level, was quantified. Scale bar, 100 µm. *p <0.05. (I) Immunostaining of NPPB on mouse heart sections of 10-month-old mice. The MFI (mean fluorescence intensity), showing NPPB expression level, was quantified. Scale bar, 100 µm. *p <0.05. (J) WGA staining on mouse heart sections of 5-month-old mice. The cell size was quantified. Scale bar, 100 µm. *p <0.05. (K) WGA staining on mouse heart sections of 10-month-old mice. The cell size was quantified. Scale bar, 100 µm. *p <0.05.

In the 3-month-old adult mice, echocardiography showed that mice exhibited a decreased ejection fraction (EF, %) and fractional shortening (FS, %) in both male and female (Figure 6A-B). We found that *Ebf1*^+/-^ mice developed cardiac hypertrophy and fibrosis (Figure 6C-G, Figure S3E). We assessed cardiac remodeling in mice at different adult stages (5 months and 10 months old) and found that NPPB, a marker of hypertrophy, was significantly upregulated in the hearts of *Ebf1*^+/-^ mice at both ages (Figure 6H-I). Additionally, cardiomyocyte size was increased in *Ebf1*^+/-^ (Figure 6J-K). These findings indicate that EBF1 depletion induces cardiac remodeling in adult mouse.

### EBF1 binds to upstream chromatins of hypertrophic genes

**Figure 7:**
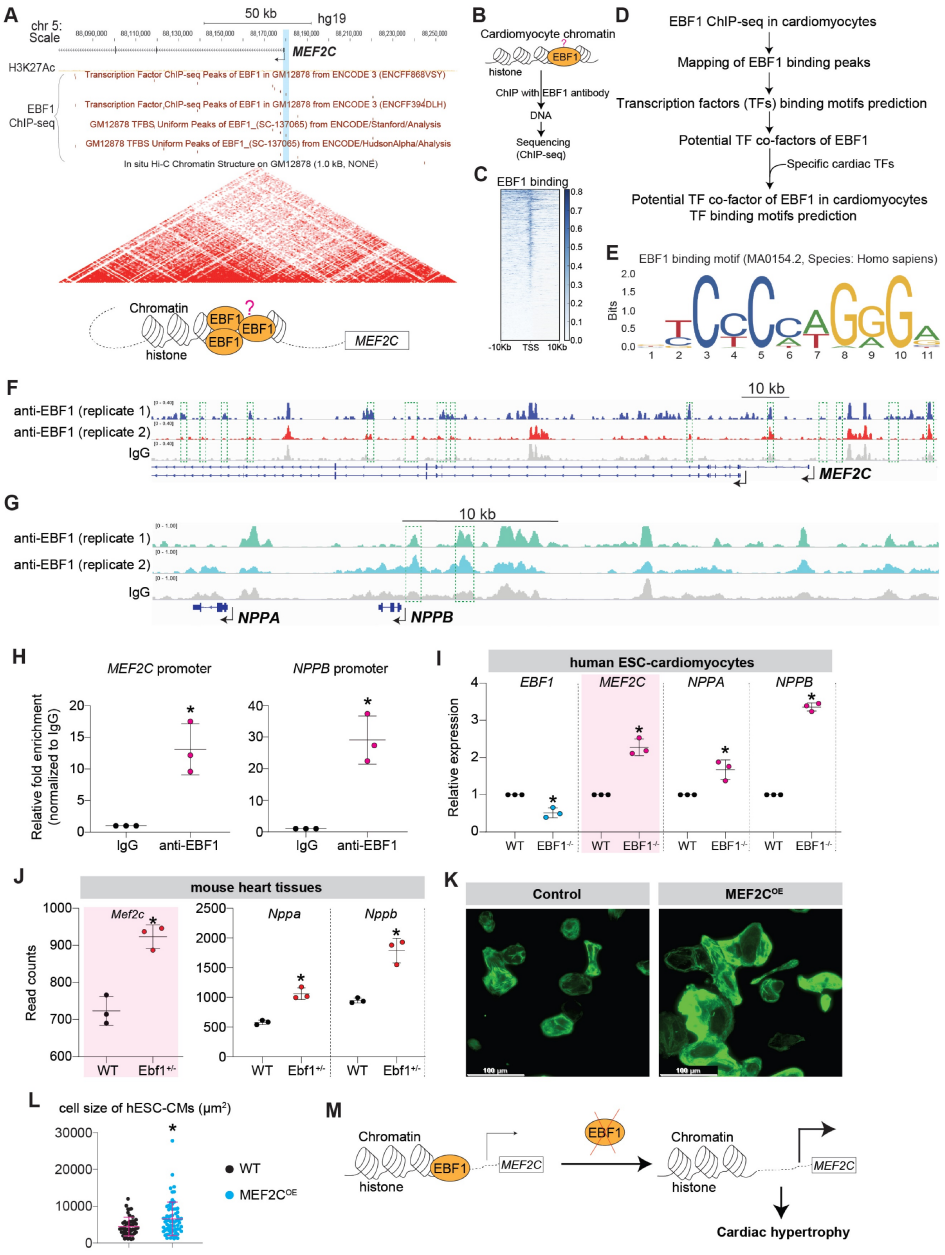
** EBF1 binds to upstream chromatin regions of hypertrophic genes.** (A) In situ Hi-C analysis showing the topologically associated domains (TADs) near *MEF2C* transcriptional start site (TSS). Conserved EBF1 binding sites near *MEF2C* TSS were presented. Blue boxes showed a conserved EBF1 binding site in *MEF2C* promoter. TADs were on chromosome 5 (chr5). Red dot showing putative EBF1 binding sites on *MEF2C* loci from several public ChIP-seq datasets of different cell types. The H3K27AC signals showing transcription activity in H1 hESCs. Data were from UCSC genome browser. (B) ChIP-seq to analyze EBF1 binding on chromatins in hESC-derived cardiomyocytes. Two replicates were performed. (C) Intensity plots showing the read densities over the peaks (± 10 kb) in profiles. TSS, near transcriptional start sites. (D-E) ChIP-seq analysis for EBF1 binding and its co-factors on genomic DNAs in cardiomyocytes. (F-G) Representative ChIP-seq peaks of EBF1 binding on hypertrophic inducer *MEF2C* (F) and hypertrophic marker (*NPPB*) (G). Highlighted green dotted boxes showing EBF1 binding regions. Anti-EBF1, antibody specifically targeting EBF1; IgG, isotype antibody as the negative control. Two biological replicates were performed for ChIP-seq. Scale in F was [0-0.40]. Scale in G was [0-1.00]. (H) ChIP-qPCR showing the binding of EBF1 on promoters of *MEF2C* and *NPPB* in hESC-derived cardiomyocytes. *p <0.05 (anti-EBF1 vs. IgG). (I) RT-qPCR showing expression levels of hypertrophic inducer (*MEF2C*) and markers (*NPPA, NPPB*) in hESC-derive cardiomyocytes. *p <0.05 (*EBF1*^-/-^ vs. WT). (J) RNA-seq analysis showing expression levels of hypertrophic inducer (*Mef2c*) and hypertrophic markers (*Nppa, Nppb*) in mouse heart tissues. *p <0.05 (*Ebf1*^+/-^ vs. WT). (K) Immunostaining showing TNNT2^+^ (green color) cardiomyocytes (CMs), which were derived from hESCs. MEF2C was overexpressed by using lentivirus. Control was the blank lentivirus. Scale bar, 100 µm. (L) Statistical analysis of cell size of hESC-CMs from (K). *p <0.05 (MEF2C^OE^ vs. Control). (M) The working model showing the potential function and mechanism mediated by EBF1 ablation in cardiac hypertrophy.

Cardiac hypertrophy results from the reactivation of hypertrophy inducers and fetal genes, such as MEF2C, NPPA and NPPB 60, 61. Analysis of published Hi-C data from the UCSC Genome Browser revealed that the genome is organized into topologically associated domains (TADs) upstream of the hypertrophic genes NPPA and NPPB (Figure S4A-B), indicating chromatin interactions at these loci. We observed several putative EBF1 binding sites within these chromatin interactions (Figure S4B). ChIP-qPCR confirmed EBF1 binding to these sites (Figure S4C-E), suggesting that the upregulation of NPPA and NPPB observed in *Ebf1* knockout mice (Figure 5G-H, Figure 6H-I) is regulated through EBF1-mediated chromatin interactions. Similarly, Hi-C data showed that the genome is organized into TADs upstream of the *MEF2C* gene, a critical hypertrophic inducer 62-66, with a conserved EBF1 binding site on the *MEF2C* promoter (Figure 7A). This suggests that EBF1 may regulate *MEF2C* expression by binding to its promoter. However, it remains unclear whether EBF1 occupies this site in cardiomyocytes and how it regulates *MEF2C* transcription.

To elucidate the EBF1-mediated regulatory mechanism in cardiomyocyte hypertrophy, we performed ChIP-seq (Figure 7B) on human cardiomyocytes derived from hESCs. Our findings highlighted that EBF1 predominantly occupied chromatin regions proximal to the transcription start sites (TSS) (Figure 7C).

The comprehensive mapping analysis of EBF1 binding peaks across genomic regions (Figure 7D-E, Figure S5A-D) uncovered EBF1 occupied the upstream genomic regions of hypertrophic inducers/genes (*MEF2C, NPPB/NPPA*) (Figure 7F-G). ChIP-qPCR confirmed EBF1 binding to the promoters of *MEF2C* and *NPPB* (Figure 7H). Thus, our data demonstrated that EBF1 directly occupied promoters of hypertrophic inducers/genes. As a result, the depletion of EBF1 reduced its binding to the promoters, resulting in increased transcription of target genes in cardiomyocytes (Figure 7I-J). Given the established role of MEF2C as a catalyst for cardiac remodeling in mouse models 9, 62, 63, 67-70, we explored whether elevated levels of MEF2C could induce cardiac remodeling in cardiomyocytes. Our investigations revealed that overexpression of MEF2C (MEF2C^OE^) effectively triggered cardiomyocyte hypertrophy (Figure 7K-L, Figure S6A-B), mirroring the outcomes observed in mouse models 9, 62, 63, 67-70. Therefore, the diminished binding of EBF1 to the* MEF2C* promoter resulted in heightened *MEF2C* transcription within cardiomyocytes, thereby contributing to the development of cardiac hypertrophy (Figure 7M).

## Discussion

The results presented in this study offer valuable insights into the role of the transcription factor EBF1 in cardiac development and cardiac remodeling. The comprehensive use of RNA-seq, ChIP-seq, and knockout strategies shed light on the diverse functions of EBF1 in regulating gene expression and cellular processes in the context of cardiac biology in both human and mouse.

Transcription factors (TFs) that are prominently expressed within cardiac tissues can profoundly influence the cardiac system. For example, in our study, we found that GATA4, NKX2-5, HAND2 and TBX5 were highly expressed in human heart tissues. Functional studies demonstrated that GATA4 25-27, NKX2-5 26, 28, HAND2 71, 72 and TBX5 73 play pivotal roles in cardiac development. Furthermore, highly expressed TFs within different regions of the heart (LV/RV/SN) identified in our study, including IRX3, KLF2, PRRX1, TFEB, RARB and ZBTB20, are well-established contributors to cardiac function 29-35. Thus, exploring TFs expression patterns within heart tissues, such as the LV/RV/SN regions as examined in this study, offers valuable insights into the mechanisms underlying cardiac function. Within this study, a specific TF, EBF1 (Early B Cell Factor 1), was identified as being highly expressed within human heart tissues. However, the functional implications of EBF1 remained unexplored within the context of human cardiac biology.

When we submitted our work, we noted a study 74 showing that *Ebf1* global knockout (KO) in mouse resulted in abnormalities in cardiac growth and differentiation. They discovered that non-cardiomyocytes express EBF1 protein, mediating non-cell-autonomous phenotypes induced by Ebf1 knockout 74. Similarly, we indeed found that mouse cardiac endothelial cells express EBF1 protein. However, our study has identified key differences. Firstly, we observed that not only some mouse cardiomyocytes but also some human cardiomyocytes express EBF1 protein. This suggests potential functions of EBF1 in cardiomyocytes within the cardiac system, which should not be excluded. Secondly, unlike that study 74, our research evaluated EBF1 expression in intact human cardiac tissues and human pluripotent stem cell-derived cardiomyocytes. We further validated EBF1 expression patterns during human cardiac development using a human pluripotent stem cell model. Our findings revealed that EBF1 deficiency impeded human mesoderm differentiation and cardiomyocyte specification, which was not reported in that study 74. Mechanistically, EBF1 could promote transcriptional expression of mesodermal and cardiogenic TFs by binding to their respective promoters. The knockout of EBF1 led to transcriptional disturbances, subsequently driving the specification of different cellular lineages. These findings underscore the critical role of EBF1 in cardiac development, providing essential knowledge for understanding of human cardiogenesis. This also facilitates a deeper comprehension of the mechanisms governing cardiac regulation. Our findings contribute significantly to the context of human cardiac biology, particularly in early cardiac development.

EBF1 has been implicated in B-cell programming and development 12-14, 75, leukemogenesis 15, cancer 16 and cell lineages formation 17, 18, 76. It serves as a pivotal TF governing B cell specification and commitment. EBF1 overexpression results in its occupancy on B cell-specific genes 77. In B cells, Ebf1-bound genes can be classified into three categories: genes activated by Ebf1, genes repressed by Ebf1, and genes that are bound by Ebf1 but are not transcriptionally active until the mature B cell stage 77. For those genes not immediately activated by EBF1, EBF1 can modify chromatin to prime target genes for future expression 77. Thus, as a TF, EBF1 can either activate, repress, or leave transcription unchanged depending on various conditions, by interacting with histones, remodeling chromatin status, or altering chromatin accessibility. However, the exact mechanisms remain elusive. Currently, whether EBF1 occupies promoters in other cellular lineages is unknown. Additionally, how EBF1 exerts control over target genes in these lineages remains unclear. In this study, we initially conducted ChIP-seq in human embryonic stem cells (hESCs), which was used as an *in vitro* model to study human cardiac development 37. This analysis revealed EBF1's direct binding to promoters of some important mesodermal and cardiogenic genes, including crucial TFs like TBXT and MESP1 involved in mesodermal differentiation, and GATA4 and NKX2-5, vital for cardiogenesis and cardiomyocyte specification. RNA-seq, RT-QPCR, flow cytometry and ChIP-seq collectively revealed that EBF1 knockout results in transcriptional repression of these genes, subsequently hindering mesoderm differentiation and cardiomyocyte specification. Hence, this study not only uncovers EBF1's role in human cardiac development but also identifies EBF1-bound genes and its regulatory impact on transcription of specific cardiac TFs.

While EBF1 is broadly expressed in various tissues and cell types such as B cells, muscle cells, and adipocytes (according to the Human Protein Atlas), its presence within cardiac lineages or heart tissues has not been definitively established. In our study, we found both EBF1 RNA and protein are expressed in cardiac lineage cells and adult human cardiac muscle tissues. ChIP-seq further unveiled EBF1 occupancy on promoters of genes linked to cardiac hypertrophy, including *MEF2C* and *NPPA/NPPB*. Genome-wide association studies (GWAS) have linked EBF1 to multiple human cardiovascular disorders, including coronary heart disease 19-22, cardiovascular metabolic disease 23 and orthostatic hypotension 24, implying its potential roles in the human cardiac system. Nonetheless, further verification is required to establish these associations. Accordingly, we postulated that EBF1 might play a role in cardiac hypertrophy. Intriguingly, Ebf1 depletion in mice led to cardiac remodeling, including hypertrophy and fibrosis. Moreover, the study's findings revealed direct EBF1 binding to upstream DNA regions of hypertrophic genes (*MEF2C, NPPA/NPPB*) in cardiomyocytes, suppressing their expression. This insight offers an explanation for the cardiac remodeling seen upon Ebf1 depletion. The observation that only WT and Ebf1^+/-^ mice, but not Ebf1^-/-^ mice, were viable at birth suggests that complete knockout of EBF1 is lethal, potentially due to the DNA damage phenotype identified in our study. These findings underscore EBF1's critical roles in the cardiac system, elucidating the underlying mechanisms and presenting a promising avenue for therapeutic intervention in cardiac remodeling.

In our study, RNA-seq demonstrated that EBF1 knockout in mouse heart tissues also led to transcriptional perturbations, which was similar with the phenotypes observed in *in vitro* cardiac development. Except for the perturbated genes in cardiomyopathy, EBF1 knockout also perturbed genes governing oxidative phosphorylation, ATP synthesis coupled electron transport and the cardiac muscle contraction. This suggests a potential role of EBF1 in cardiac metabolism, warranting further investigation. Furthermore, EBF1 knockout was found to decrease the expression levels of certain cardiac ion channel genes, indicating its potential regulation of the conduction and contraction within the heart. Previous report highlighted EBF1's significance in proper formation of the cardiac ventricular conduction system (in an abstract of 2016 American Heart Association late-breaking Basic Science) 78. In the report, Ebf1 was observed to decrease expression levels of Nkx2-5, Gata4, Hand1, Irx3, and Irx5, all critical TFs for cardiogenesis 78. Ebf1 was found to bind directly to the Nkx2-5 promoter, influencing proper Nkx2-5 expression during the development of the cardiac ventricular conduction system in mice 78. An additional investigation has identified distinct subtypes of ventricular cardiomyocytes in humans 79, with one subset demonstrating elevated levels of both EBF1 and EBF2. This finding suggests EBF1's potential involvement in the cardiac contraction system. However, many aspects of EBF1's roles within the cardiac system, as well as its functions in the human heart, remain unclear. Our study revealed that EBF1 knockout results in reduced expression levels of key cardiogenic TFs, such as MESP1, GATA4, and NKX2-5, during human cardiac development. We found that EBF1 directly bond on promoters of these TFs, which is important for their proper expression. Interestingly, in adult mice, we have also observed that depleting Ebf1 significantly downregulates certain ion channel genes, except for NKX2-5 (also reported in the previous abstract 78). Our finding implies that Ebf1's influence extends to not only cardiac conduction and contraction system but also cardiac development, and also emphasizes the potential comprehensive scope of EBF1's roles within the cardiac system.

Some TFs, crucial for cardiomyocyte specification and differentiation, such as GATA4 10 and MEF2C 11, have also been implicated in cardiac remodeling 3-5. This supports to the notion that regulatory networks governing cardiomyocyte specification may overlap with those underlying cardiac remodeling. Consequently, investigating TF functions in cardiomyocyte specification could serve as an alternative avenue for comprehending the pathogenesis of cardiac hypertrophy. The cardiac hypertrophy was initially identified several decades ago 80, 81. However, the intricate mechanisms through which TFs mediate cardiac hypertrophy remain elusive. Accumulating evidence highlights the essential roles of TFs in cardiac hypertrophy and their potential as therapeutic targets 9, 11, 53, 82, 83. For example, GATA4 is a zinc finger-containing transcription factor that plays an essential role in promoting cardiac development and cardiomyocyte differentiation 84, 85. Cardiac-specific deletion of Gata4 revealed its requirement for hypertrophy, compensation, and myocyte viability 53. Transcription factor NFAT3, activated by Ca2^+^/calmodulin-dependent protein phosphatase calcineurin, was also sufficient to evoke myocardial hypertrophy 81. Additionally, transcription factor MEF2C, activated by CaM kinase signaling pathway, induced cardiac hypertrophy as well 62. In addition to GATA, MEF2 and NFAT families, other transcription factors, such as UBF1 86, ATF3 87, T-Cdk9 88, XBP1 89, MITF 90, HSF1 91, C/EBP 92, KLF11 93 and KLF5/BTEB2 94 have been implicated in regulating cardiac hypertrophy. This collectively indicates that TF-mediated transcriptional reprogramming events are pivotal for in both cardiac development and cardiac hypertrophy. However, while this knowledge is substantial, more exploration into various TFs is essential to pave the way for novel therapies targeting human cardiac hypertrophy 95. Our study's findings not only illuminate the role of EBF1 in controlling cardiac development but also unveil its involvement in cardiac hypertrophy. This suggests the intriguing possibility that regulatory networks governing cardiac development might intersect with those driving cardiac remodeling (hypertrophy and fibrosis).

## Conclusions

In summary, we elucidate the functions of EBF1 in both cardiac development and cardiac remodeling in both human and mouse. We find that EBF1 regulates the transcription of downstream target genes by directly binding to promoters or upstream chromatins. These findings in our study unravel the multifaceted contributions of EBF1 in cardiac biology, from cardiac development to disease, shedding light on potential therapeutic avenues.

### Limitations

Although we have elucidated the functions and underlying mechanisms of EBF1 in both human and mouse cardiac systems, including its roles in regulating cardiac development and cardiac remodeling, several unknown questions persist in this study. EBF1 may be involved in long-range chromatin interaction, whether and how it regulates chromatin as a potential 3D chromatin organizer in cardiac system are unclear. TF binding motif analyses of our ChIP-seq data discovered that NKX2-5 might be the co-factor interacting with EBF1 to regulate target genes transcription. However, whether it can interact with other TFs or epigenetic regulators to orchestrate the transcription on promoter is unclear. Furthermore, while EBF1 may have influence on oxidative phosphorylation and the cardiac conduction system, the precise mechanisms governing these effects remain enigmatic. Thus, further investigations are waiting to address these questions, which will deepen our understanding in the future.

## Materials and Methods

### Human heart tissues

All human samples from healthy donor and HCM patients were obtained according to institutional guidelines (Guangdong Provincial People's Hospital, Guangdong Academy of Medical Sciences, Guangdong, Guangzhou, CHINA). Written, informed consent was obtained in advance from donors. Tissues were dissected under an inverted microscope and stored at -80°C for RNA-seq, RT-QPCR and section. The collection and usage about the human heart tissues were conducted according to the principles of the Declaration of Helsinki and was approved by the Research Ethical Committee of the Guangdong Provincial People's Hospital.

### Cell culture

Human induced pluripotent stem cells (hiPSCs) were reprogrammed from live cells isolated from urine of healthy donor in Guangzhou Institutes of Biomedicine and Health, Chinese Academy of Sciences (Guangzhou, CHINA) according to the published method 96. H9 human ESCs (hESCs) was gifted by Professor Xiaohong Li in Guangdong Provincial People's Hospital (CHINA). All human pluripotent stem cells (hPSCs), including H9 hESCs and hiPSCs, were maintained on matrigel-coated plate in mTesR1 medium (STEM CELL Technologies, Canada). Cells were routinely passaged by ReLeSR™ Human PSC Selection & Passaging Reagent (STEM CELL Technologies, Canada). Cells have been maintained in our laboratory since then using the above conditions and are routinely tested to be free of mycoplasma.

### Cardiac differentiation and Cardiomyocyte enrichment

H9 human ESCs (hESCs) were routinely maintained on matrigel-coated plate in mTesR1 medium. For cardiac differentiation, hESCs were initially dissociated and re-seeded on matrigel-coated 12-well plates. When hESCs achieved 100% confluence, they were differentiated towards cardiomyocytes based on previous protocol 97. Briefly, cells were incubated with RPMI1640 medium (Gibco, USA) plus B27(minus insulin) (Gibco, USA) with CHIR99021 (6 µM) for 24 hours. After that, cells were maintained in RPMI1640 medium plus B27 (minus insulin) for 48 hours. Then 5 μM XAV939 was added at day 3 in RPMI1640 medium plus B27 (minus insulin) for more 48 hours. Cells were maintained in the RPMI1640 medium with B27 (minus insulin) starting from day 7. The medium was renewed every 2 days. On day 14, post-differentiated beating cells were subjected to lactate selection 98 for 4 days, in which the selection medium was changed every 2 days. The lactate selection medium was DEME (no glucose) (Gibco, USA) plus with lactate (final concentration 4 mM). After lactate selection, beating cells were maintained in DMEM (no glucose) medium plus 10% fetal bovine serum (FBS) (Gibco, USA). On day 50, beating cardiomyocytes were used for hypertrophy associated experiments. For cardiomyocyte specification and differentiation experiments, day 0 to day 7 differentiated cells were collected according to the experimental design. All chemicals were purchased from Tocris Bioscience (USA).

### RNA extraction

Total RNAs from cells were extracted by miRNeasy mini kit (Qiagen, Germany) or RNeasy Mini Kit (Qiagen, Germany). Total RNAs from human and mouse heart tissues were extracted by using TRIzol (Qiagen, Germany) and stainless-steel bead kit with shaker, and purified by using miRNeasy mini kit (Qiagen, Germany).

### Real-time RT-PCR

CDNAs were produced using a High-Capacity RNA-to-cDNA kit (Life Technologies, USA), and Real-time PCR was performed using Power SYBR Green Master Mix (Thermo Fisher Scientific, USA) in a 7500 Real-Time PCR System (Life Technologies, USA) according to the manufacturer's instructions. All PCR reactions were performed in triplicate, normalized to the internal control genes GAPDH or beta-actin, and analyzed by using the comparative 2^-ΔΔCt^ method. Primer sequences used for real-time PCR were shown in supplemental information.

### Immunostaining

Cells and heart sections were fixed in 4% paraformaldehyde for 10 minutes at room temperature (RT). Permeabilization and blocking were performed 1× PBS with 10% BSA and 1% Triton X-100 for 30 minutes at RT. Cells were incubated with primary antibody at 4 °C overnight. Secondary antibody was applied for 1 hour at 37 °C. After nuclear staining with DAPI (Invitrogen, USA), the images were captured by confocal-laser microscopy (Leica, Germany).

### Flow cytometry

Flow cytometry was performed according to our protocol 37. Briefly, cells were harvested by using 0.25% trypsin for 5 minutes at 37°C. The dissociated cells were fixed by 4% PFA for 10 minutes at room temperature and subsequently washed three times with 1× PBS. Cells were incubated with first antibody in blocking buffer (1× PBS plus 2% goat serum and 0.1% saponin). Then cells were washed with 1× PBS for 3 times, following with incubation with second antibody in blocking buffer at 37°C for 1 hour. Flow cytometry evaluation was carried out with BD LSRII cytometer (Becton Dickinson, USA) or Attune NxT Flow Cytometer (Thermo Fisher Scientific, USA). Data were analyzed by FlowJo (Treestar).

### CRISPR/Cas9 mediated gene knockout

gRNA targeting EBF1 was designed based on CRISPR design platform (http://crispr.mit.edu/). Human EBF1 gene was knocked out by single gRNA in H9 hESCs. For gRNA oligo, self-complementary oligos were purchased from Invitrogen. EBF1 gRNA, targeting the exon sequence, was cloned into the lentiCRISPRv2-puro lentivirus vector (Addgene) 99. Virus package was performed on HEK293T cells. H9 hESCs were infected by the virus and puromycin was used to screen positive clones. Stable EBF1 knockout cell lines were routinely maintained in mTesR1 medium.

### Virus package, production and infection

Virus was produced by co-transfecting lentivirus vector together with psPAX2 and pMD2.G into HEK293T cells by using X-tremeGENE™ 9 DNA Transfection Reagent (Roche). 24 hours after transfection, the medium was replaced with fresh DMEM (high glucose) medium with 10% FBS. Supernatant containing viruses was collected twice, on 48 hours and 72 hours after transfection, separately. Virus was stored at 4 °C for no more than one week or at -80 °C for more than one week. Before infection, virus was filtered through a 0.22-μm filter. Lenti-viruses were purified by the Lenti-X™ Concentrator kit (Takara Bio, Japan). The purified viruses were responded in 1 × PBS and stored in -80 °C. For virus infection of human ESCs, cells were seeded on 6-well plates pre-coated with Matrigel. 24 hours later, cells were infected with virus for 5 hours in 37 °C incubator. After that, the medium was changed back to mTeSR1 medium for overnight. The same infection was repeated the next day. After 24h of second infection, puromycin was added to mTesR1 medium to screen positive cell clones.

### Mouse model

The animal protocols used in this study were approved by the Institutional Review Board (IRB) at Guangdong Provincial People's Hospital and Guangdong Academy of Medical Sciences (Guangzhou, China). And all animal experiments were conducted in accordance with the Institutional Review Board (IRB) at Guangdong Provincial People's Hospital and Guangdong Academy of Medical Sciences (Guangzhou, China). The male and female C57BL/6J mice of WT and Ebf1 knockout were purchased from Cyagen Biosciences (Guangzhou, CHINA) and used throughout this study. All mice were housed under specific pathogen-free (SPF) conditions with standard chow and bedding with 12 hours day/night cycle according to institutional protocols.

### Mouse Echocardiography and Electrocardiogram (ECG)

Echocardiography was performed using the Vevo 2100 ultrasound system (VisualSonics, Canada) equipped with a MS-550 linear-array probe working at a central frequency of 40 MHz. After the animals were anesthetized with 3.0 % (v/v) isoflurane carried by pure oxygen, they were placed at supine position on a pre-warmed platform at around 37°C. Then, hair removal cream was used to remove hair on chest and abdomen. Subsequently, the anesthesia was not maintained and echocardiography was performed under conscious condition. The eye gel was used to prevent ocular dehydration. Needle probes attached to ECG leads embedded in the imaging platform were subcutaneously inserted to each limb for ECG. ECG was monitored and maintained during the whole echocardiography procedure. Left ventricular (LV) geometry and function were evaluated using M-mode from parasternal short-axis. LV anterior (LVAW) and posterior (LVPW) wall thickness and internal dimensions (LVID) were evaluated at the M-mode during systole (s) and diastole (d). LV ejection fraction (EF) was calculated from the volumes (Vol), which are computed according to the Teichholz formula. And the fractional shortening (FS) was also calculated. Data was transferred to an offline computer and analyzed with Vevo 2100 software (VisualSonics, Canada) by a technician blinded to the study groups. ECG data were recorded and analyzed using the MedLab-U/4C501H equipped with the ECG Analysis Module (SHENJIAN company, Shanghai, China). Peak amplitudes and intervals of electrocardiogram (ECG) were determined by the equipment. After echocardiography and ECG, mice were euthanized via cervical dislocation under anesthesia and hearts were dissected for other experiments.

### Histology

Heart sections, HE (hematoxylin and eosin) staining and Masson's trichrome staining were performed by the servicebio company (Wuhan, China). Briefly, the heart tissues were fixed with z-fix (Anatech Ltd, USA) overnight, dehydrated in 70% ethanol, and embedded in paraffin and 7 µm sections were prepared. Hematoxylin and eosin (HE), WGA and EBF1 staining were also performed by servicebio company. The tissues were fixed with 4% paraformaldehyde in 1× PBS overnight and dehydrated through sequential ethanol washes. The tissues were then embedded in paraffin and 7 µm sections were prepared for HE, WGA and EBF1 staining. The section images were obtained by a Leica upright Microscope.

### Immunohistochemistry

Heart tissues were embedded in paraffin, cut to 7 μm, and placed onto slides. After the endogenous peroxidase was blocked with 0.6% hydrogen peroxide in methanol and the nonspecific background was reduced with 10% normal goat serum, slides were incubated with antibody at room temperature for overnight. Subsequently, slides were washed with tap water and incubated with a secondary horseradish peroxidase conjugate at room temperature for 1 hour. Slides were then washed again with tap water and incubated with freshly prepared 3-amino-9-ethylcarbazole (Sigma, USA) dissolved in N, N-dimethylformamide, sodium acetate and hydrogen peroxide for 15 minutes. Specificity of the antibody was confirmed by substitution with nonimmune goat serum. Finally, all slides were examined by Leica microscopy and photographed.

### Chromatin immunoprecipitation (ChIP)

H9 hESCs or H9-derived cardiomyocytes were cultured in P10 plate in mTesR1 medium or DMEM (no glucose) plus 10% FBS, respectively. ChIP was performed according to manuals of truChIP™ Chromatin Shearing Kit (Covaris, PN 520154, USA) and EZ-Magna ChIP™ A/G Chromatin Immunoprecipitation Kit (Millipore, 17-10086, USA). Briefly, cells were fixed with methanol-free formaldehyde included in the truChIP™ Chromatin Shearing Kit (Covaris, PN 520154, USA). Then chromatin DNAs of cell lysis were sheared using truChIP™ Chromatin Shearing Kit according its manual by the ME220 Focused-ultrasonicator (Covaris, USA). The sheared chromatin DNAs were incubated with anti-EBF1 antibody (anti-IgG or 1% Input as the control), then purified by using EZ-Magna ChIP™ A/G Chromatin Immunoprecipitation Kit (Millipore, 17-10086, USA). Chromatin DNAs pulled down by anti-EBF1 and anti-IgG and the 1% Input were for QPCR to evaluate EBF1 binding or directly submitted to Experimental Department in Novogene (Novogene bioinformatics Technology Co. Ltd, CHINA) for sequencing.

### ChIP-seq data analysis

All ChIP-seq data analysis were performed by Novogene bioinformatics Technology Co. Ltd (CHINA). ChIP-seq raw data (raw reads) of fastq format were firstly processed through in-house perl scripts. Clean data (clean reads) were obtained by removing reads containing adapter, reads containing ploy-N and low-quality reads from raw data. Index of the reference genome was built using BWA v0.7.12 and clean reads were aligned to the reference genome using BWA mem v 0.7.12. After mapping reads to the reference genome, the MACS2 version 2.1.0 (model-based analysis of ChIP-seq) peak finding algorithm was used to identify regions of ChIP enrichment over background. A q value threshold of enrichment of 0.05 was used for all data sets. Then, the distribute of chromosome distribution, peak width, fold enrichment, significant level and peak summit number per peak were all displayed. Different peak analysis was based on the fold enrichment of peaks of different experiments. A peak was determined as different peak when the odds ratio between two groups (anti-EBF1 vs. anti-IgG) was more than 2. Using the same method, genes associated with different peaks were identified and also do GO and KEGG enrichment analysis. At least two biological replicates were applied for ChIP-seq.

### RNA-seq

All of library preparation and the RNA-seq were performed in Experimental Department in Novogene (Novogene bioinformatics Technology Co. Ltd). A total amount of 3 μg RNA per sample was used as input material for the RNA sample preparations. Sequencing libraries were generated using NEBNext® UltraTM RNA Library Prep Kit for Illumina® (NEB, USA) following manufacturer's maunals and index codes were added to attribute sequences to each sample. Briefly, mRNA was purified from total RNA using poly-T oligo-attached magnetic beads. Fragmentation was carried out using divalent cations under elevated temperature in NEBNext First Strand Synthesis Reaction Buffer(5X). First strand cDNA was synthesized using random hexamer primer and M-MuLV Reverse Transcriptase (RNase H^-^). Second strand cDNA synthesis was subsequently performed using DNA Polymerase I and RNase H. Remaining overhangs were converted into blunt ends via exonuclease/polymerase activities. After adenylation of 3' ends of DNA fragments, NEBNext Adaptor with hairpin loop structure were ligated to prepare for hybridization. In order to select cDNA fragments of preferentially 150~200 bp in length, the library fragments were purified with AMPure XP system (Beckman Coulter, Beverly, USA). Then 3 μl USER Enzyme (NEB, USA) was used with size-selected, adaptor-ligated cDNA at 37°C for 15 min followed by 5 min at 95 °C before PCR. Then PCR was performed with Phusion High-Fidelity DNA polymerase, Universal PCR primers and Index (X) Primer. At last, PCR products were purified (AMPure XP system) and library quality was assessed on the Agilent Bioanalyzer 2100 system. The clustering of the index-coded samples was performed on a cBot Cluster Generation System using TruSeq PE Cluster Kit v3-cBot-HS (Illumia) according to the manufacturer's instructions. After cluster generation, the library preparations were sequenced on an Illumina NovaSeq 6000 platform and 125 bp/150 bp paired-end reads were generated.

### RNA-seq data analysis

All RNA-seq data analysis was conducted by Experimental Department in Novogene (Novogene bioinformatics Technology Co. Ltd, China).

(1) Quality control: Raw data (raw reads) of fastq format were firstly processed through in-house perl scripts. In this step, clean data (clean reads) were obtained by removing reads containing adapter, reads containing ploy-N and low-quality reads from raw data. At the same time, Q20, Q30 and GC content the clean data were calculated. All the downstream analyses were based on the clean data with high quality.

(2) Reads mapping to the reference genome: Reference genome and gene model annotation files were downloaded from genome website directly. Index of the reference genome was built using STAR and paired-end clean reads were aligned to the reference genome using STAR (v2.5.1b). STAR used the method of Maximal Mappable Prefix (MMP) which can generate a precise mapping result for junction reads.

(3) Quantification of gene expression level: HTSeq v0.6.0 was used to count the reads numbers mapped to each gene. And then FPKM of each gene was calculated based on the length of the gene and reads count mapped to this gene. FPKM, expected number of Fragments Per Kilobase of transcript sequence per millions base pairs sequenced, considers the effect of sequencing depth and gene length for the reads count at the same time, and is currently the most commonly used method for estimating gene expression levels.

(4) Differential expression analysis: (For DESeq2 with biological replicates) Differential expression analysis of at least two conditions/groups was performed using the DESeq2 R package (1.10.1). DESeq2 provide statistical routines for determining differential expression in digital gene expression data using a model based on the negative binomial distribution. The resulting P-values were adjusted using the Benjamini and Hochberg's approach for controlling the false discovery rate. Genes with an adjusted P-value <0.05 found by DESeq2 were assigned as differentially expressed.

### ChIP-qPCR

ChIP was performed by using EZ-Magna ChIP™ G - Chromatin Immunoprecipitation Kit (Millipore, USA) according to the manual. Briefly, about 1 × 10^7^cells were fixed by 1% formaldehyde at room temperature for 10 minutes. After fixation, 1 × glycine was used to quench unreacted formaldehyde at room temperature for 5 minutes. Cells were collected and washed by 1 × PBS, then lysed by cell lysis buffer. Cell pellet was collected, then lysed by nuclear lysis buffer. Nuclear chromatin was sonicated and pulled down by EBF1 antibody. And the DNAs were purified by using spin column in kit. Normal mouse/rabbit IgG antibodies (Millipore EZ-Magna ChIP kit) were used as negative control, respectively. ChIP-qPCR signals were calculated as fold enrichment of 1% Input or non-specific antibody (isotype IgG antibodies) signals with at less three technical triplicates. Each specific antibody ChIP sample was normalized to its isotype IgG antibody-ChIP-signals obtained in the same sample. Standard deviations (SD) were calculated from technical triplicates and represented as error bars. Primer sequences used for real-time PCR were shown in supplemental information.

### Functional enrichment analysis

Gene Ontology (GO) enrichment analysis of differentially expressed genes was implemented by the clusterProfiler R package, in which gene length bias was corrected. GO terms with corrected P value less than 0.05 were considered significantly enriched by differential expressed genes. KEGG was performed for large-scale molecular datasets (http://www.genome.jp/kegg/). ClusterProfiler R package was used to test the statistical enrichment of differential expression genes in KEGG pathways by Experimental Department in Novogene (CHINA). GO analysis was also performed on GENEONTOLOGY (http://geneontology.org/docs/go-enrichment-analysis/). Signaling pathway analysis was run on Reactome. Protein-protein interaction analysis was performed by the online tool STRING: functional protein association networks (https://string-db.org/).

### Quantification and statistical analysis

All statistical analyses were performed using the Graphpad Prism 8. Data were represented as mean ± SD of biological replicate experiments; individual data points were also shown. The statistical significance was evaluated using Student's unpaired *t* test (two-tailed) (comparison between two groups) or One-Way ANOVA (comparison for more than two groups). A p value of less than 0.05 was considered statistically significant.

## Supplementary Material

Supplementary figures.

Supplementary tables.

## Abbreviations

EBF1: EBF Transcription Factor 1
TF: Transcription Factor
hPSCs: human pluripotent stem cells
hiPSCs: human induced pluripotent stem cells
hESCs: human embryonic stem cells
CMs: cardiomyocytes
CRISPR: Clustered regularly interspaced short palindromic repeat
RV: right ventricle
LV: left ventricle
SN: sinoatrial node
TUNEL: Terminal deoxynucleotidyl transferase dUTP nick end labeling
RNA-seq: RNA Sequencing
ChIP-seq: chromatin immunoprecipitation (ChIP) assays with sequencing
GO: Gene Ontology
KEGG: Kyoto Encyclopedia of Genes and Genomes
