# Supplementary material for: Transcriptome analysis reveals EBF1 ablation-induced injuries in cardiac system
Source: Theranostics. 2024 Aug 12;14(12):4894–915. doi: 10.7150/thno.92060 (PMC11373621; doi:10.7150/thno.92060)

**Supplemental Figure legends:**

**Figure S1. RNA-seq analysis of LV, RV, SN and hiPSCs and the expression of EBF1 in different cell types**

(A) Pearson correlation analysis for all 4 samples.

(B-F) Subcluster analysis based on gene expression profiling of LV, RV, SN and hiPSCs. Gene expression levels in the subcluster were up-regulated in LV/RV/SN (B), hiPSCs (C), SN (D), RV (E) or LV (F). Self-organizing maps (SOM) analysis was applied to the RNA-seq data to identify patterns of gene expression across 4 groups (LV, RV, SN and hiPSCs). GO analysis was run by the THE GENE ONTOLOGY RESOURCE (http://geneontology.org/).

(G) The *in vitro* model of human cardiac development. RNA-seq was performed to evaluate gene expression patterns during cardiac development. Cell samples on day 0 (hiPSCs), day 3 (mesodermal cells) and day 5 (cardiac progenitor cells) were collected for RNA-seq.

(H) Gene Ontology (GO) analysis of up-regulated genes during *in vitro* human cardiac development from (G).

(I) Heat map showing expression levels of cardiogenic TFs, which are reported to be crucial for cardiogenesis, during cardiac development.

(J) Statistic analysis of EBF1 protein expression pattern during cardiac development of hESCs. *$p < 0.05$.

(K) The *in vitro* model of human cardiac development and cardiomyocyte differentiation. Cardiomyocytes could be purified by using lactate. Flow cytometry was used to analyze EBF1 expression in non-purified cardiomyocytes and purified cardiomyocytes.

(L) Immunostaining of EBF1 in different mouse cardiac cell types, including cardiomyocytes ($CTNT^+$ cells), endothelial cells ($CD31^+$ cells) and smooth muscle cells ($\alpha$-$SMA^+$ cells).

**Figure S2. EBF1 knockout inhibits mesoderm differentiation and cardiomyocyte specification**

(A) CRISPR/Cas9 to knock out EBF1 mediated by lentivirus vector (lentiCRISPRv2).

(B-C) RT-qPCR showing expression levels of *EBF1* (B), and the genes controlling mesoderm differentiation (*TBXT, MESP1)* (C). *p <0.05 (EBF1 gRNA2 vs. Control; EBF1 gRNA3 vs. Control).

(D) Flow cytometry showing the percentage of $\alpha$-$SMA^+$ cells on day 7 post cardiac differentiation. *p <0.05 ($EBF1^{-/-}$ vs. WT).

**Figure S3. EBF1, expressed in both human and mouse heart tissues, is potentially involved in cardiac function**

(A) *EBF1* is expressed in different human tissues. Data was from NCBI database.

(B) *Ebf1* is expressed in different mouse tissues. Data was from NCBI database.

(C-D) Potential phenotypes of *Ebf1* knockout in mouse model. Magnetic Resonance Imaging (MRI) showed some cardiac phenotypes in mouse model (D). Data were from the public database (informatics.jax.org/marker/MGI:95275).

(E) Western blot showing the protein expression of cardiac fibrosis marker (COL1A1) and cardiac hypertrophy marker (NPPB) in 3-month-old mouse heart tissues.

**Figure S4. EBF1 binds on upstream chromatin of hypertrophic markers NPPA/NPPB**

(A-B) In situ Hi-C analysis showing the topologically associated domains (TADs) near *NPPB* transcriptional start site (TSS). Conserved and putative EBF1 binding sites near NPPB were presented. TADs were on chromosome 5 (chr5). Highlighted colorful boxes and lines showing the chromatin interactions. Several putative EBF1 binding sites, localized on upstream regions of NPPB loci, were found in the TADs. Data were from UCSC genome browser.

(C-E) ChIP-qPCR to evaluate the binding of EBF1 on *NPPB* chromatin. Different primers targeting to different regions were designed (C). ChIP-qPCR were conducted to detect binding of EBF1 on distal regions (D) and promoter regions (E). $p^* < 0.05$ (anti-EBF1 vs. anti-IgG).

**Figure S5. EBF1 binding motif analysis**

(A) EBF1 potential binding motifs.

(B) The matrix of potential EBF1 binding motifs.

(C) NKX2-5 potential binding motif on EBF1 bound regions. NKX2-5 was enriched after analysis of ChIP-seq peaks by using transcription factors (TFs) binding motifs. This indicated that NKX2-5 may be a potential co-factor of EBF1.

(D) Protein-protein interaction analysis of EBF1 and putative EBF1-interacting transcription factors (TFs) in cardiac system. The TFs were from the output of binding motif analyses in EBF1 bound regions in ChIP-seq. This data demonstrated that EBF1 may be involved in cardiac regulatory networks, by potentially interacting with some crucial cardiogenic TFs (such as NKX2-5).

**Figure S6. MEF2C promotes cardiac hypertrophy in hESC-derived cardiomyocytes**

(A) Immunostaining showing the protein expression of cardiomyocyte marker (TNNT2, green color) and cardiac hypertrophy marker (NPPB, red color). MEF2C$^{OE}$, MEF2C overexpression by lentivirus infection. Control, blank lentivirus infection. Scale bar, 100 μm.

(B) The percentage of $NPPB^+$ hESC-derived cardiomyocytes (CMs). *p <0.05 ($MEF2C^{OE}$ vs. Control).

# Figure 1

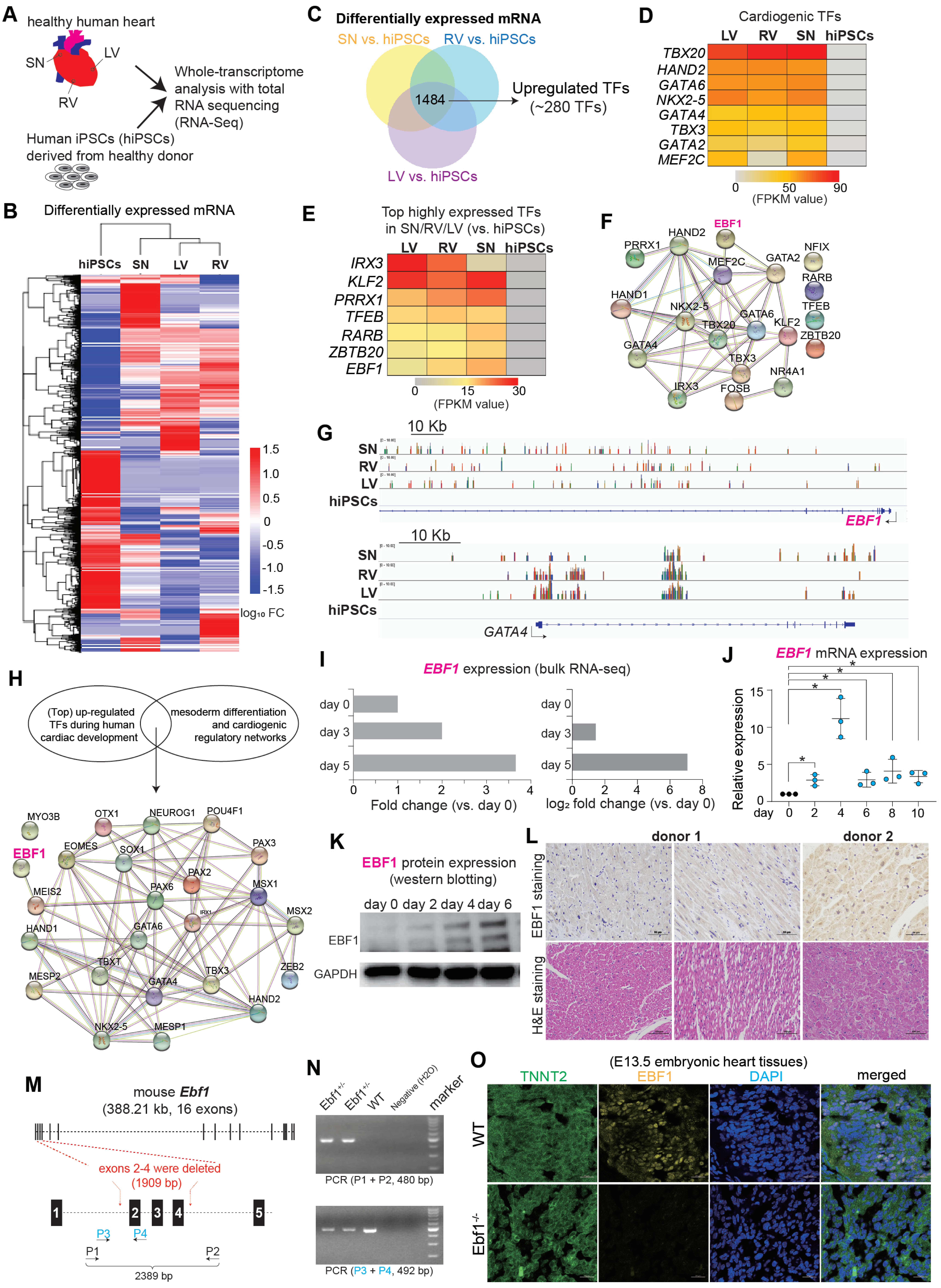

A
healthy human heart
SN
LV
RV
Whole-transcriptome analysis with total RNA sequencing (RNA-Seq)
Human iPSCs (hiPSCs) derived from healthy donor
B
Differentially expressed mRNA
hiPSCs SN LV RV
1.5
1.0
0.5
0
-0.5
-1.0
-1.5
log10 FC
C
Differentially expressed mRNA
SN vs. hiPSCs
RV vs. hiPSCs
LV vs. hiPSCs
1484
Upregulated TFs (~280 TFs)
D
Cardiogenic TFs
LV RV SN hiPSCs
TBX20
HAND2
GATA6
NKX2-5
GATA4
TBX3
GATA2
MEF2C
0 50 90
(FPKM value)
E
Top highly expressed TFs in SN/RV/LV (vs. hiPSCs)
LV RV SN hiPSCs
IRX3
KLF2
PRRX1
TFEB
RARB
ZBTB20
EBF1
0 15 30
(FPKM value)
F
EBF1
HAND2
PRRX1
NFIX
MEF2C
GATA2
RARB
HAND1
NKX2-5
GATA6
TFEB
TBX20
KLF2
ZBTB20
GATA4
TBX3
NR4A1
IRX3
FOSB
G
10 Kb
SN
RV
LV
hiPSCs
EBF1
10 Kb
SN
RV
LV
hiPSCs
GATA4
H
(Top) up-regulated TFs during human cardiac development
mesoderm differentiation and cardiogenic regulatory networks
MYO3B
OTX1
NEUROG1
POU4F1
EBF1
EOMES
SOX1
PAX3
PAX2
MEIS2
PAX6
MSX1
IRX1
MSX2
HAND1
GATA6
TBXT
ZEB2
MESP2
TBX3
GATA4
HAND2
NKX2-5
MESP1
I
EBF1 expression (bulk RNA-seq)
day 0
day 3
day 5
0 1 2 3 4
Fold change (vs. day 0)
0 2 4 6 8
log2 fold change (vs. day 0)
J
EBF1 mRNA expression
Relative expression
0 5 10 15
day 0 2 4 6 8 10
K
EBF1 protein expression (western blotting)
day 0 day 2 day 4 day 6
EBF1
GAPDH
L
donor 1
donor 2
EBF1 staining
H&E staining
M
mouse Ebf1
(388.21 kb, 16 exons)
exons 2-4 were deleted (1909 bp)
1 2 3 4 5
P3 P4
P1 P2
2389 bp
N
Ebf1+/- Ebf1+/- WT Negative (H2O) marker
PCR (P1 + P2, 480 bp)
PCR (P3 + P4, 492 bp)
O
(E13.5 embryonic heart tissues)
TNNT2
EBF1
DAPI
merged
WT
Ebf1-/-

**Figure 2**

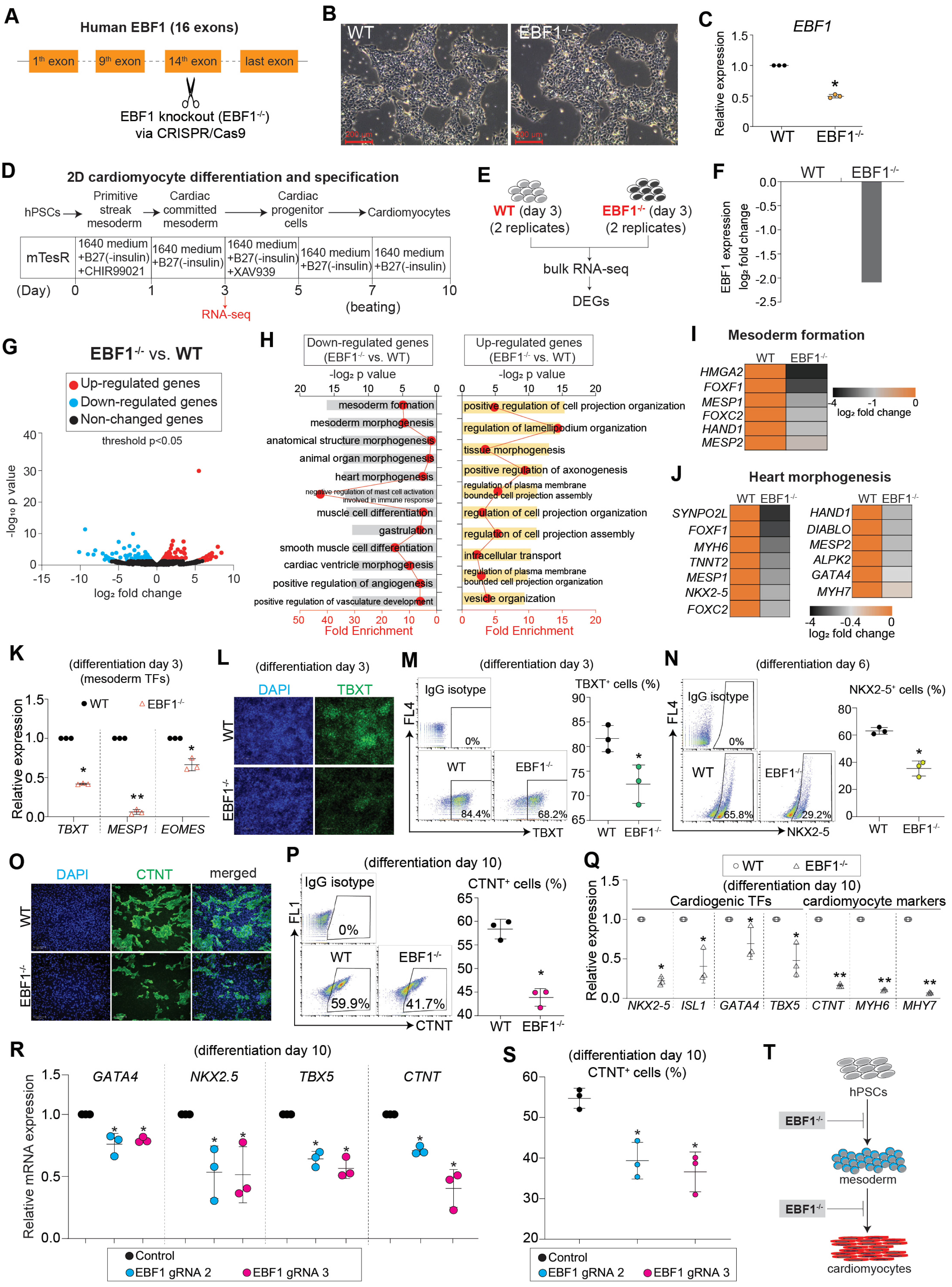
A
Human EBF1 (16 exons)
1th exon
9th exon
14th exon
last exon
EBF1 knockout (EBF1-/-)
via CRISPR/Cas9
B
WT
EBF1-/-
C
EBF1
Relative expression
1.5
1.0
0.5
0
WT
EBF1-/-
D
2D cardiomyocyte differentiation and specification
hPSCs
Primitive streak mesoderm
Cardiac committed mesoderm
Cardiac progenitor cells
Cardiomyocytes
mTesR
1640 medium +B27(-insulin) +CHIR99021
1640 medium +B27(-insulin)
1640 medium +B27(-insulin) +XAV939
1640 medium +B27(-insulin)
1640 medium +B27(-insulin)
(Day) 0 1 3 5 7 10
RNA-seq
(beating)
E
WT (day 3)
(2 replicates)
EBF1-/- (day 3)
(2 replicates)
bulk RNA-seq
DEGs
F
WT
EBF1-/-
EBF1 expression
log2 fold change
0.0
-0.5
-1.0
-1.5
-2.0
-2.5
G
EBF1-/- vs. WT
Up-regulated genes
Down-regulated genes
Non-changed genes
threshold p<0.05
-log10 p value
log2 fold change
H
Down-regulated genes (EBF1-/- vs. WT)
Up-regulated genes (EBF1-/- vs. WT)
-log2 p value
mesoderm formation
mesoderm morphogenesis
anatomical structure morphogenesis
animal organ morphogenesis
heart morphogenesis
negative regulation of mast cell activation involved in immune response
muscle cell differentiation
gastrulation
smooth muscle cell differentiation
cardiac ventricle morphogenesis
positive regulation of angiogenesis
positive regulation of vasculature development
positive regulation of cell projection organization
regulation of lamellipodium organization
tissue morphogenesis
positive regulation of axonogenesis
regulation of plasma membrane bounded cell projection assembly
regulation of cell projection organization
regulation of cell projection assembly
intracellular transport
regulation of plasma membrane bounded cell projection organization
vesicle organization
Fold Enrichment
I
Mesoderm formation
WT
EBF1-/-
HMGA2
FOXF1
MESP1
FOXC2
HAND1
MESP2
log2 fold change
J
Heart morphogenesis
SYNPO2L
FOXF1
MYH6
TNNT2
MESP1
NKX2-5
FOXC2
HAND1
DIABLO
MESP2
ALPK2
GATA4
MYH7
log2 fold change
K
(differentiation day 3)
(mesoderm TFs)
WT
EBF1-/-
Relative expression
TBXT
MESP1
EOMES
L
(differentiation day 3)
DAPI
TBXT
WT
EBF1-/-
M
(differentiation day 3)
IgG isotype
0%
WT
84.4%
EBF1-/-
68.2%
FL4
TBXT
TBXT+ cells (%)
WT
EBF1-/-
N
(differentiation day 6)
IgG isotype
0%
WT
65.8%
EBF1-/-
29.2%
FL4
NKX2-5
NKX2-5+ cells (%)
WT
EBF1-/-
O
DAPI
CTNT
merged
WT
EBF1-/-
P
(differentiation day 10)
IgG isotype
0%
WT
59.9%
EBF1-/-
41.7%
FL1
CTNT
CTNT+ cells (%)
WT
EBF1-/-
Q
WT
EBF1-/-
(differentiation day 10)
Cardiogenic TFs
cardiomyocyte markers
Relative expression
NKX2-5
ISL1
GATA4
TBX5
CTNT
MYH6
MHY7
R
(differentiation day 10)
GATA4
NKX2.5
TBX5
CTNT
Relative mRNA expression
Control
EBF1 gRNA 2
EBF1 gRNA 3
S
(differentiation day 10)
CTNT+ cells (%)
Control
EBF1 gRNA 2
EBF1 gRNA 3
T
hPSCs
EBF1-/-
mesoderm
EBF1-/-
cardiomyocytes

# Figure 3

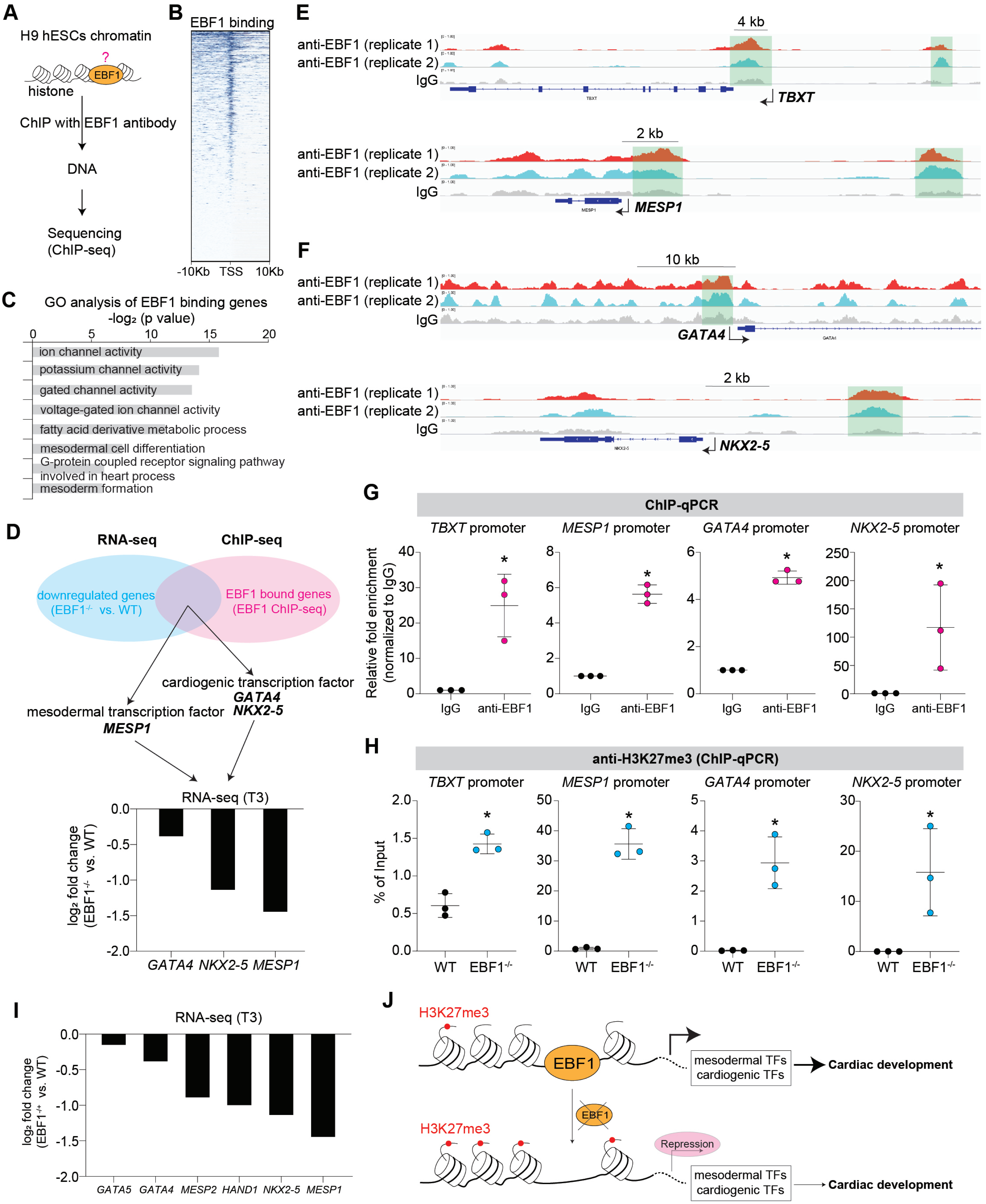

A
H9 hESCs chromatin
EBF1
histone
ChIP with EBF1 antibody
DNA
Sequencing (ChIP-seq)
B
EBF1 binding
-10Kb TSS 10Kb
C
GO analysis of EBF1 binding genes
-log2 (p value)
ion channel activity
potassium channel activity
gated channel activity
voltage-gated ion channel activity
fatty acid derivative metabolic process
mesodermal cell differentiation
G-protein coupled receptor signaling pathway involved in heart process
mesoderm formation
D
RNA-seq
ChIP-seq
downregulated genes (EBF1-/- vs. WT)
EBF1 bound genes (EBF1 ChIP-seq)
cardiogenic transcription factor GATA4 NKX2-5
mesodermal transcription factor MESP1
RNA-seq (T3)
log2 fold change (EBF1-/- vs. WT)
GATA4 NKX2-5 MESP1
E
anti-EBF1 (replicate 1)
anti-EBF1 (replicate 2)
IgG
4 kb
TBXT
2 kb
MESP1
F
10 kb
GATA4
2 kb
NKX2-5
G
ChIP-qPCR
TBXT promoter
MESP1 promoter
GATA4 promoter
NKX2-5 promoter
Relative fold enrichment (normalized to IgG)
IgG anti-EBF1
H
anti-H3K27me3 (ChIP-qPCR)
% of Input
WT EBF1-/-
I
RNA-seq (T3)
log2 fold change (EBF1-/+ vs. WT)
GATA5 GATA4 MESP2 HAND1 NKX2-5 MESP1
J
H3K27me3
EBF1
mesodermal TFs cardiogenic TFs
Cardiac development
Repression

# Figure 4

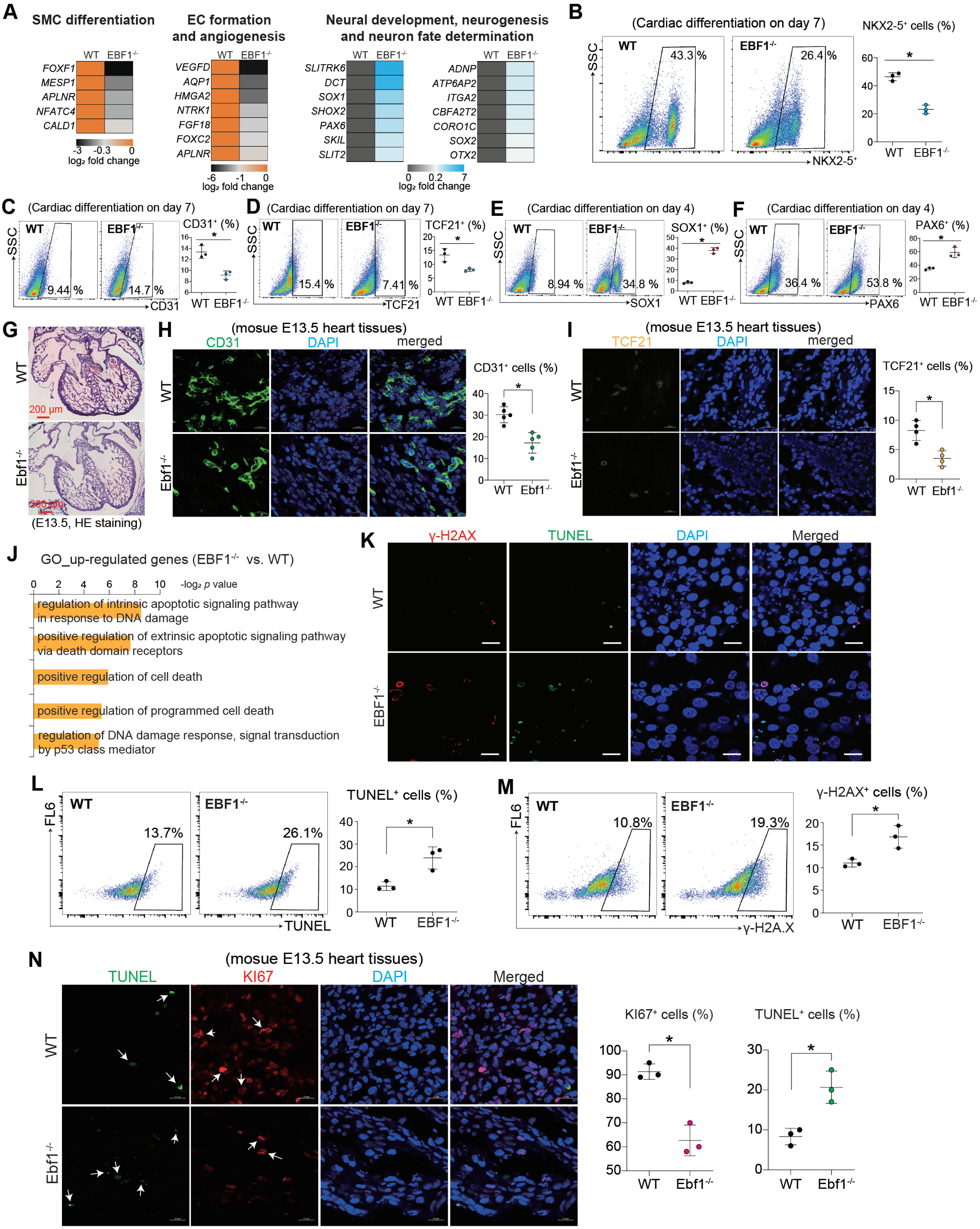
A
SMC differentiation
EC formation and angiogenesis
Neural development, neurogenesis and neuron fate determination
WT EBF1-/-
FOXF1
MESP1
APLNR
NFATC4
CALD1
VEGFD
AQP1
HMGA2
NTRK1
FGF18
FOXC2
SLITRK6
DCT
SOX1
SHOX2
PAX6
SKIL
SLIT2
ADNP
ATP6AP2
ITGA2
CBFA2T2
CORO1C
SOX2
OTX2
log2 fold change
B
(Cardiac differentiation on day 7)
NKX2-5+ cells (%)
SSC
NKX2-5+
43.3 %
26.4 %
C
(Cardiac differentiation on day 7)
CD31+ (%)
9.44 %
14.7 %
CD31
D
(Cardiac differentiation on day 7)
TCF21+ (%)
15.4 %
7.41 %
TCF21
E
(Cardiac differentiation on day 4)
SOX1+ (%)
8.94 %
34.8 %
SOX1
F
(Cardiac differentiation on day 4)
PAX6+ (%)
36.4 %
53.8 %
PAX6
G
WT
Ebf1-/-
200 µm
(E13.5, HE staining)
H
(mosue E13.5 heart tissues)
CD31
DAPI
merged
CD31+ cells (%)
I
(mosue E13.5 heart tissues)
TCF21
DAPI
merged
TCF21+ cells (%)
J
GO_up-regulated genes (EBF1-/- vs. WT)
-log2 p value
regulation of intrinsic apoptotic signaling pathway in response to DNA damage
positive regulation of extrinsic apoptotic signaling pathway via death domain receptors
positive regulation of cell death
positive regulation of programmed cell death
regulation of DNA damage response, signal transduction by p53 class mediator
K
γ-H2AX
TUNEL
DAPI
Merged
L
FL6
13.7%
26.1%
TUNEL+ cells (%)
TUNEL
M
10.8%
19.3%
γ-H2AX+ cells (%)
γ-H2A.X
N
(mosue E13.5 heart tissues)
TUNEL
KI67
DAPI
Merged
KI67+ cells (%)
TUNEL+ cells (%)
WT
Ebf1-/-

# Figure 5

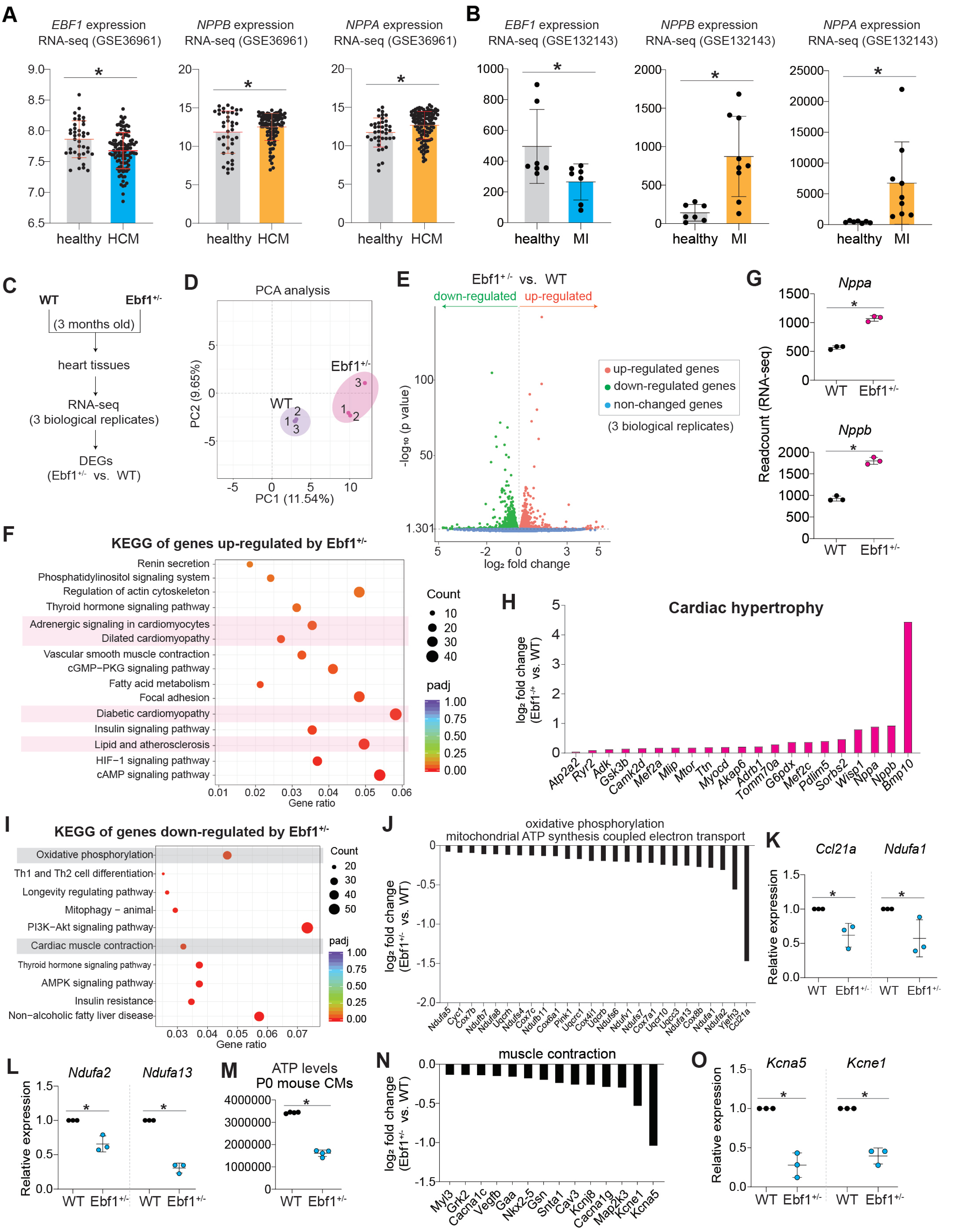
A
EBF1 expression RNA-seq (GSE36961)
NPPB expression RNA-seq (GSE36961)
NPPA expression RNA-seq (GSE36961)
healthy HCM
B
EBF1 expression RNA-seq (GSE132143)
NPPB expression RNA-seq (GSE132143)
NPPA expression RNA-seq (GSE132143)
healthy MI
C
WT
Ebf1+/-
(3 months old)
heart tissues
RNA-seq
(3 biological replicates)
DEGs
(Ebf1+/- vs. WT)
D
PCA analysis
PC2 (9.65%)
PC1 (11.54%)
WT
Ebf1+/-
E
Ebf1+/- vs. WT
down-regulated
up-regulated
up-regulated genes
down-regulated genes
non-changed genes
(3 biological replicates)
-log10 (p value)
log2 fold change
G
Nppa
Nppb
Readcount (RNA-seq)
WT Ebf1+/-
F
KEGG of genes up-regulated by Ebf1+/-
Renin secretion
Phosphatidylinositol signaling system
Regulation of actin cytoskeleton
Thyroid hormone signaling pathway
Adrenergic signaling in cardiomyocytes
Dilated cardiomyopathy
Vascular smooth muscle contraction
cGMP−PKG signaling pathway
Fatty acid metabolism
Focal adhesion
Diabetic cardiomyopathy
Insulin signaling pathway
Lipid and atherosclerosis
HIF−1 signaling pathway
cAMP signaling pathway
Gene ratio
Count
padj
H
Cardiac hypertrophy
log2 fold change (Ebf1-/+ vs. WT)
Atp2a2 Ryr2 Adk Gsk3b Camk2d Mef2a Mlip Mtor Ttn Myocd Akap6 Adrb1 Tomm70a G6pdx Mef2c Pdlim5 Sorbs2 Wisp1 Nppa Nppb Bmp10
I
KEGG of genes down-regulated by Ebf1+/-
Oxidative phosphorylation
Th1 and Th2 cell differentiation
Longevity regulating pathway
Mitophagy − animal
PI3K−Akt signaling pathway
Cardiac muscle contraction
Thyroid hormone signaling pathway
AMPK signaling pathway
Insulin resistance
Non−alcoholic fatty liver disease
Gene ratio
Count
padj
J
oxidative phosphorylation
mitochondrial ATP synthesis coupled electron transport
log2 fold change (Ebf1+/- vs. WT)
Ndufa5 Cyc1 Cox7b Ndufb7 Ndufa8 Uqcrh Ndufs4 Cox7c Ndufb11 Cox6a1 Pink1 Uqcrc1 Cox4i1 Uqcrb Ndufs6 Ndufv1 Ndufs7 Cox7a1 Uqcr10 Uqcc3 Ndufa13 Cox8b Ndufa1 Ndufa2 Ylefn3 Ccl21a
K
Ccl21a
Ndufa1
Relative expression
WT Ebf1+/-
L
Ndufa2
Ndufa13
Relative expression
WT Ebf1+/-
M
ATP levels
P0 mouse CMs
WT Ebf1+/-
N
muscle contraction
log2 fold change (Ebf1+/- vs. WT)
Myl3 Grk2 Cacna1c Vegfb Gaa Nkx2-5 Gsn Snta1 Cav3 Kcnj8 Cacna1g Map2k3 Kcne1 Kcna5
O
Kcna5
Kcne1
Relative expression
WT Ebf1+/-

# Figure 6

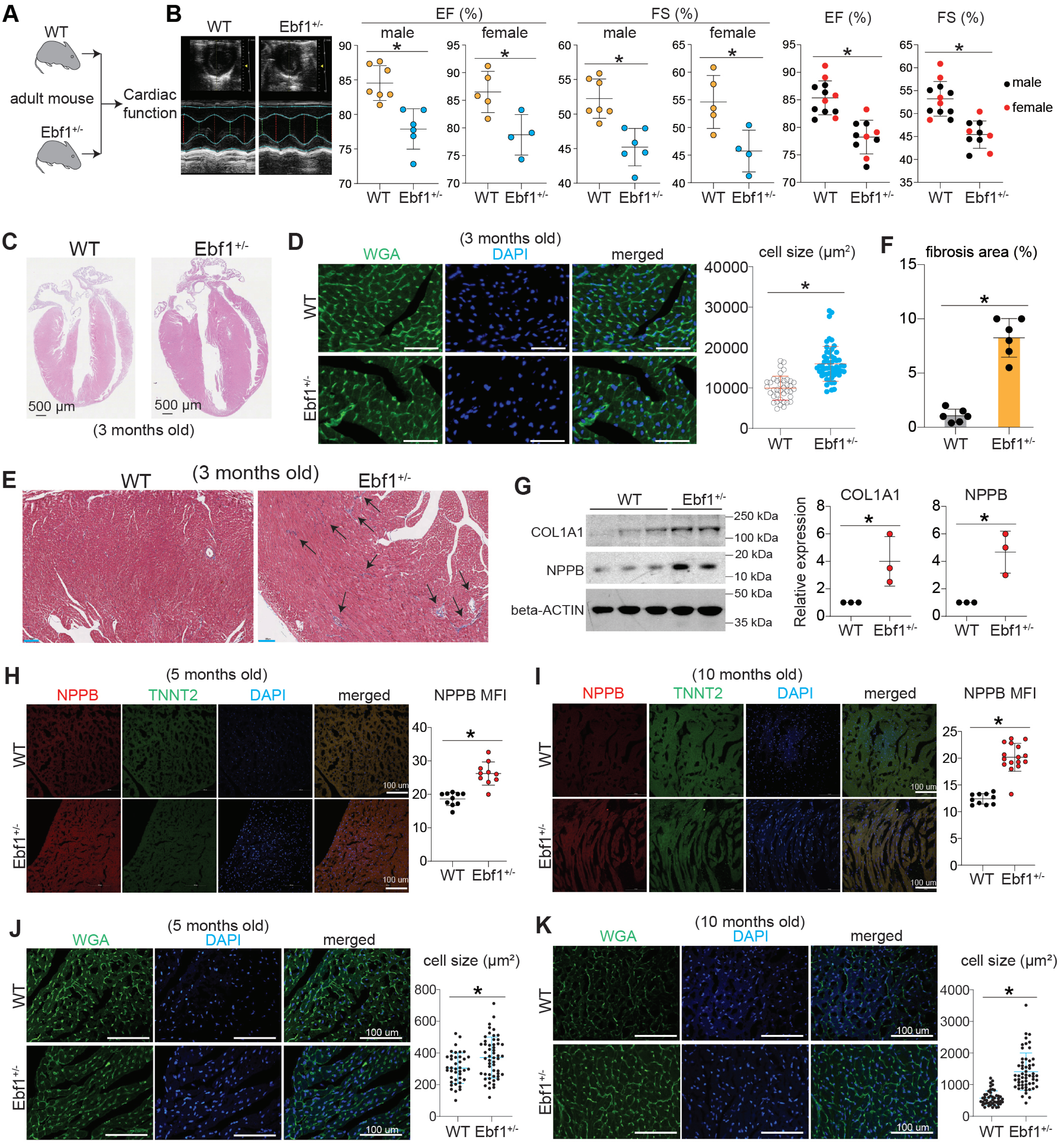
A
WT
adult mouse
Ebf1+/-
Cardiac function
B
WT
Ebf1+/-
EF (%)
male
female
FS (%)
male
female
EF (%)
FS (%)
male
female
C
WT
Ebf1+/-
500 μm
500 μm
(3 months old)
D
(3 months old)
WGA
DAPI
merged
cell size (μm2)
F
fibrosis area (%)
E
WT
(3 months old)
Ebf1+/-
G
WT
Ebf1+/-
COL1A1
NPPB
beta-ACTIN
250 kDa
100 kDa
20 kDa
10 kDa
50 kDa
35 kDa
Relative expression
COL1A1
NPPB
H
(5 months old)
NPPB
TNNT2
DAPI
merged
NPPB MFI
100 um
I
(10 months old)
NPPB
TNNT2
DAPI
merged
NPPB MFI
J
(5 months old)
WGA
DAPI
merged
cell size (μm²)
K
(10 months old)
WGA
DAPI
merged
cell size (μm²)

Figure 7

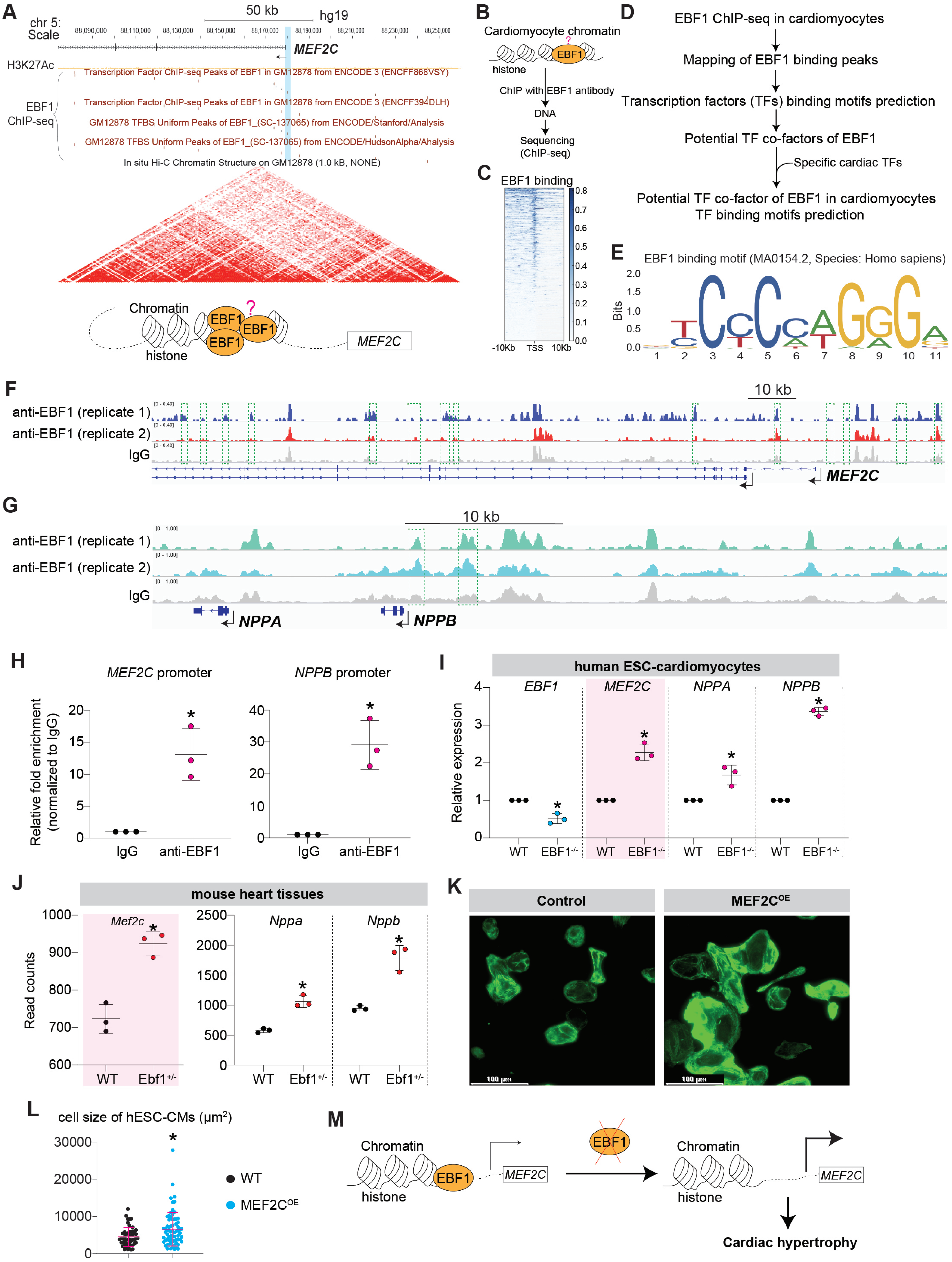

A
chr 5:
Scale
50 kb
hg19
MEF2C
H3K27Ac
EBF1 ChIP-seq
Transcription Factor ChIP-seq Peaks of EBF1 in GM12878 from ENCODE 3 (ENCFF868VSY)
Transcription Factor ChIP-seq Peaks of EBF1 in GM12878 from ENCODE 3 (ENCFF394DLH)
GM12878 TFBS Uniform Peaks of EBF1_(SC-137065) from ENCODE/Stanford/Analysis
GM12878 TFBS Uniform Peaks of EBF1_(SC-137065) from ENCODE/HudsonAlpha/Analysis
In situ Hi-C Chromatin Structure on GM12878 (1.0 kB, NONE)
Chromatin
EBF1
?
histone
MEF2C
B
Cardiomyocyte chromatin
histone
ChIP with EBF1 antibody
DNA
Sequencing (ChIP-seq)
C
EBF1 binding
-10Kb
TSS
10Kb
D
EBF1 ChIP-seq in cardiomyocytes
Mapping of EBF1 binding peaks
Transcription factors (TFs) binding motifs prediction
Potential TF co-factors of EBF1
Specific cardiac TFs
Potential TF co-factor of EBF1 in cardiomyocytes
TF binding motifs prediction
E
EBF1 binding motif (MA0154.2, Species: Homo sapiens)
Bits
F
anti-EBF1 (replicate 1)
anti-EBF1 (replicate 2)
IgG
10 kb
MEF2C
G
anti-EBF1 (replicate 1)
anti-EBF1 (replicate 2)
IgG
10 kb
NPPA
NPPB
H
MEF2C promoter
NPPB promoter
Relative fold enrichment (normalized to IgG)
IgG
anti-EBF1
I
human ESC-cardiomyocytes
EBF1
MEF2C
NPPA
NPPB
Relative expression
WT
EBF1-/-
J
mouse heart tissues
Mef2c
Nppa
Nppb
Read counts
WT
Ebf1+/-
K
Control
MEF2COE
100 µm
L
cell size of hESC-CMs (µm2)
WT
MEF2COE
M
Chromatin
EBF1
histone
MEF2C
Cardiac hypertrophy

# Supplemental Figure 1

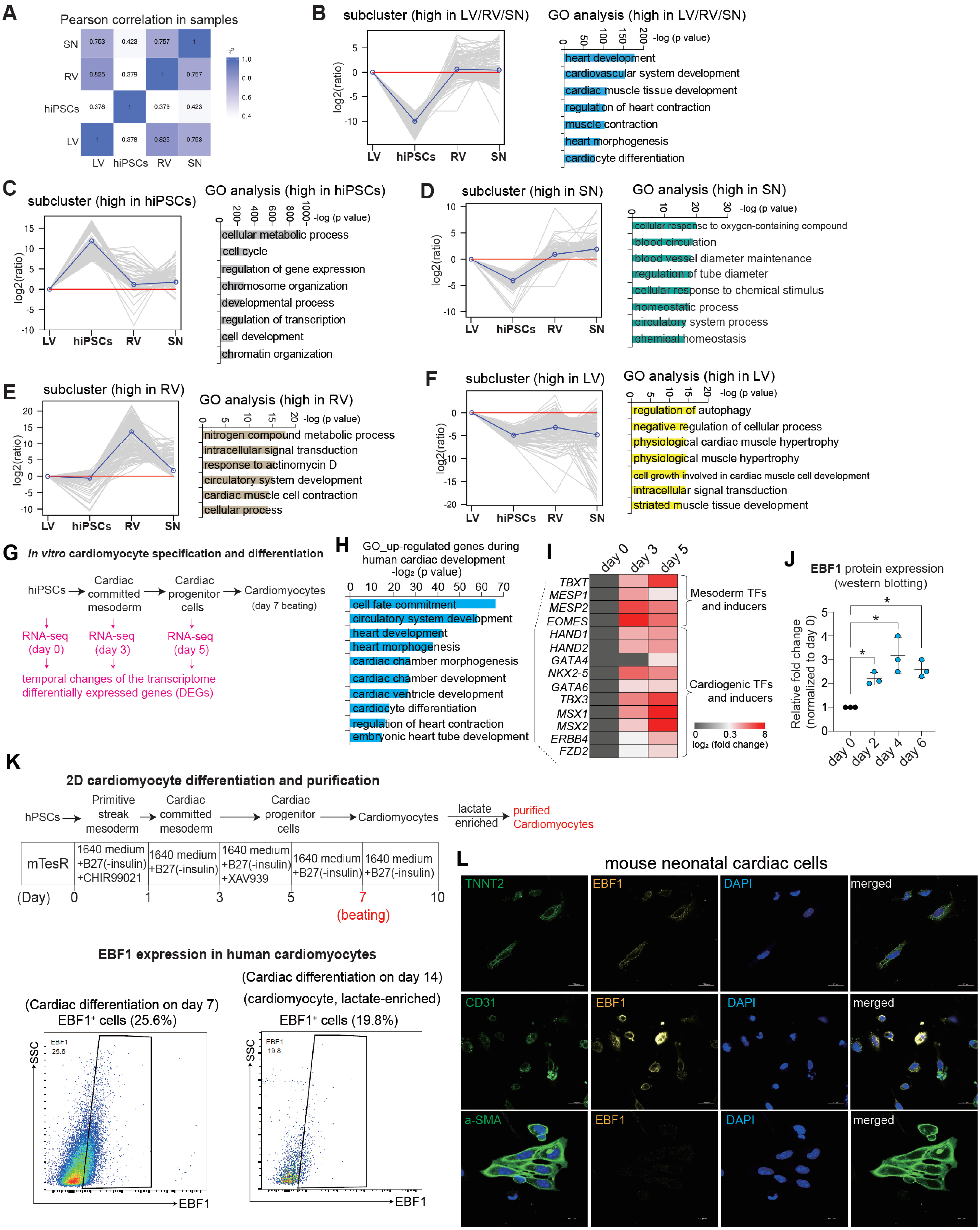

**Supplemental Figure 2**

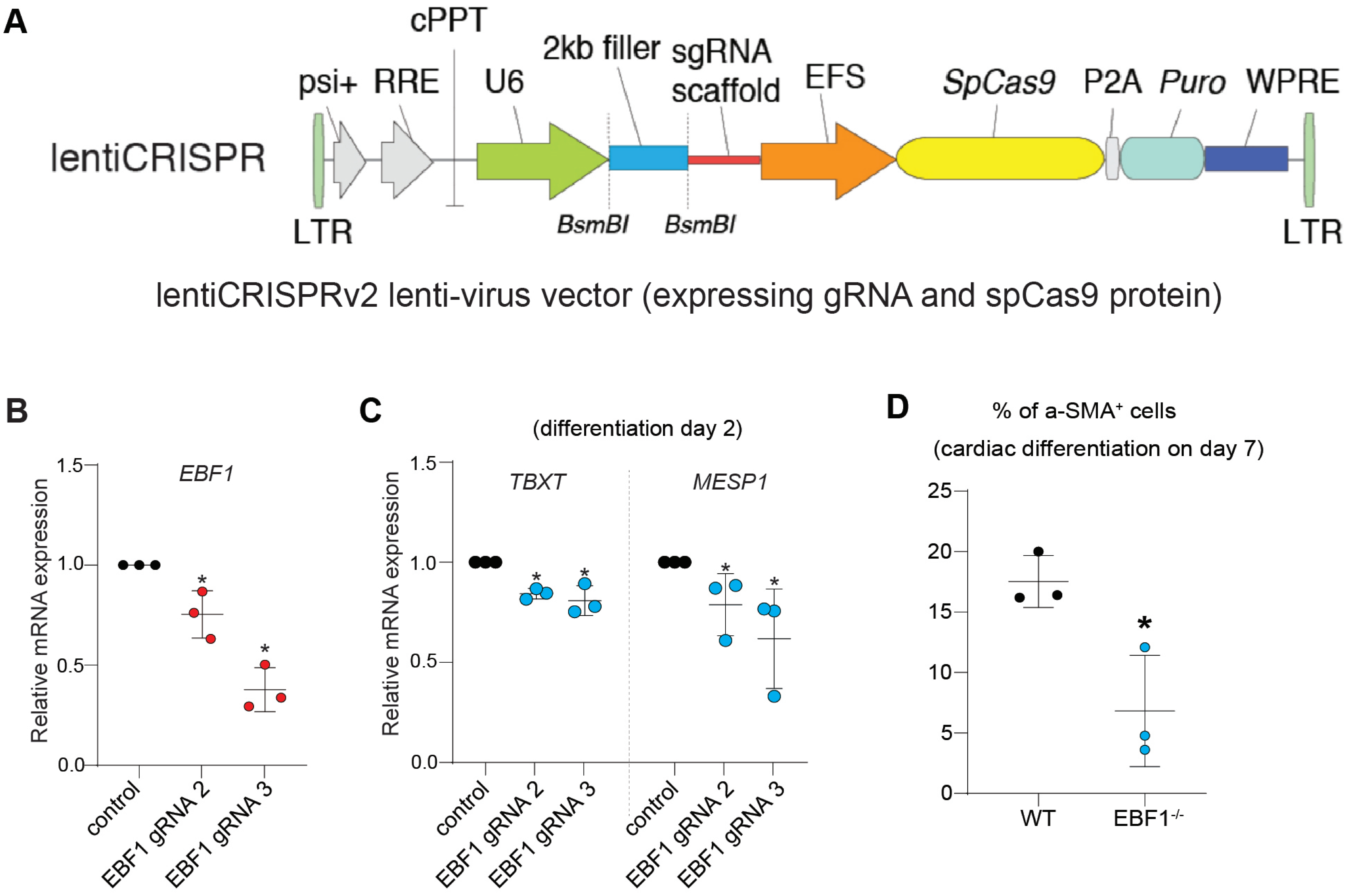

# Supplemental Figure 3

A

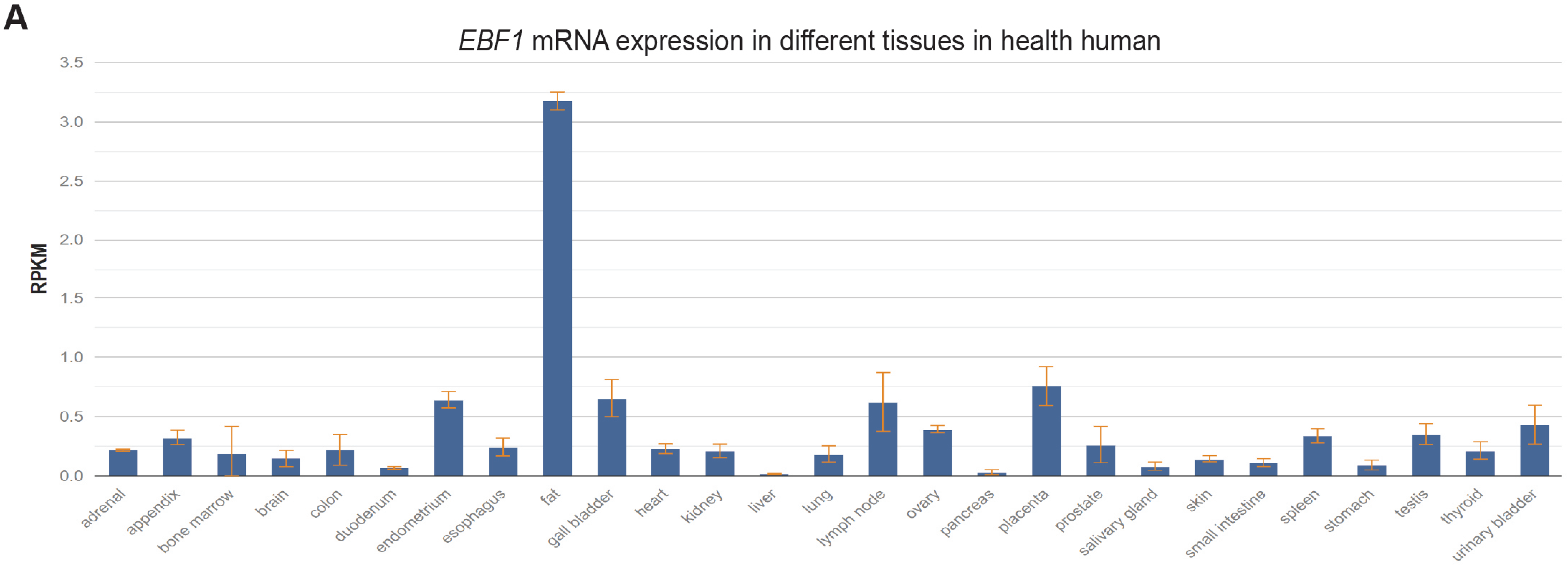


B

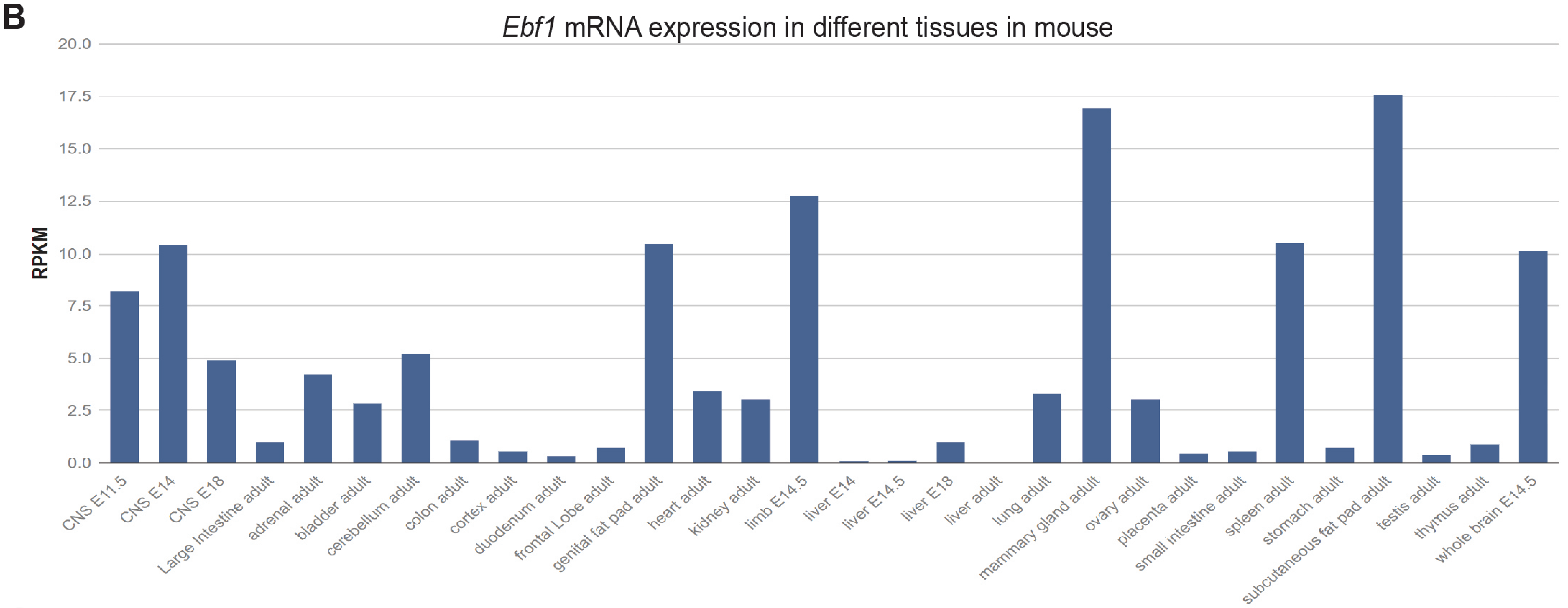


C

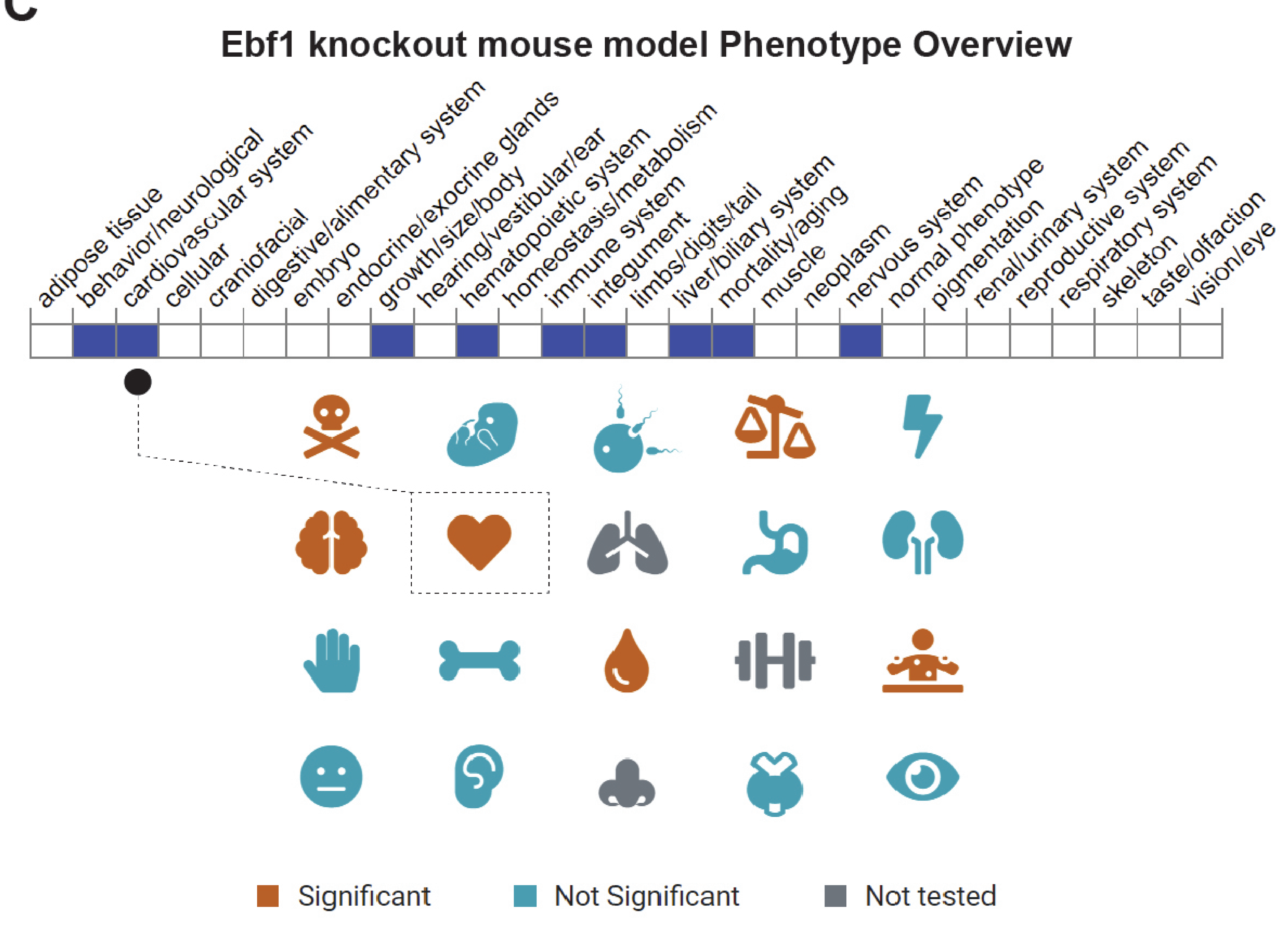


D

**Cardiac phenotype overview of Ebf1 knockout mouse (vs. WT)**

| Genotype | Phenotype | Sex | Life Stage | P Value |
|---|---|---|---|---|
| -/- | preweaning lethality incomplete penetrance | male/female | early adult | 0 |
| +/- | abnormal heart morphology | male/female | late adult | 0 |
| +/- | small heart | female | late adult | 0 |
| +/- | enlarged heart | male | late adult | 0 |

**E18.5 embryonic hearts**

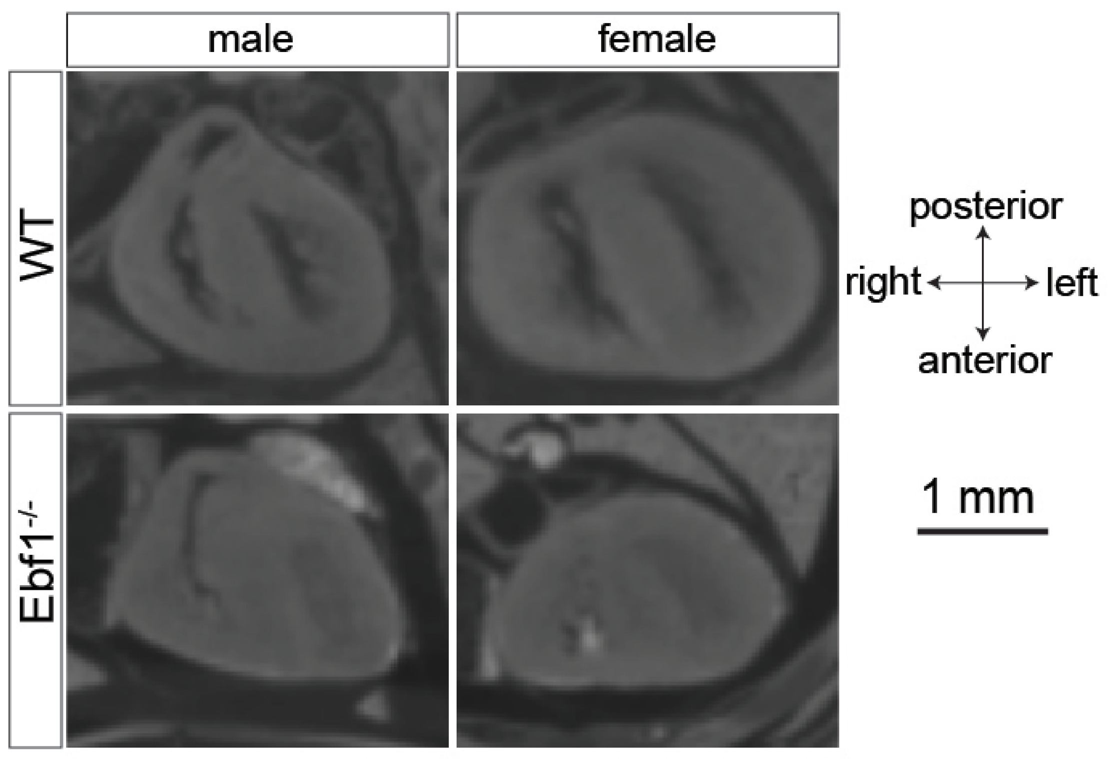


E

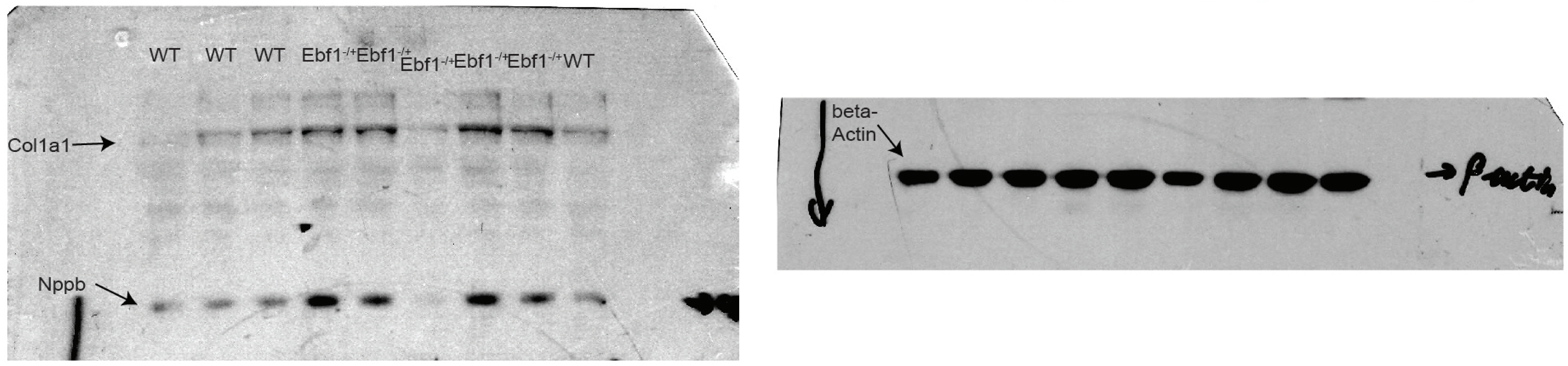

## Supplemental Figure 4

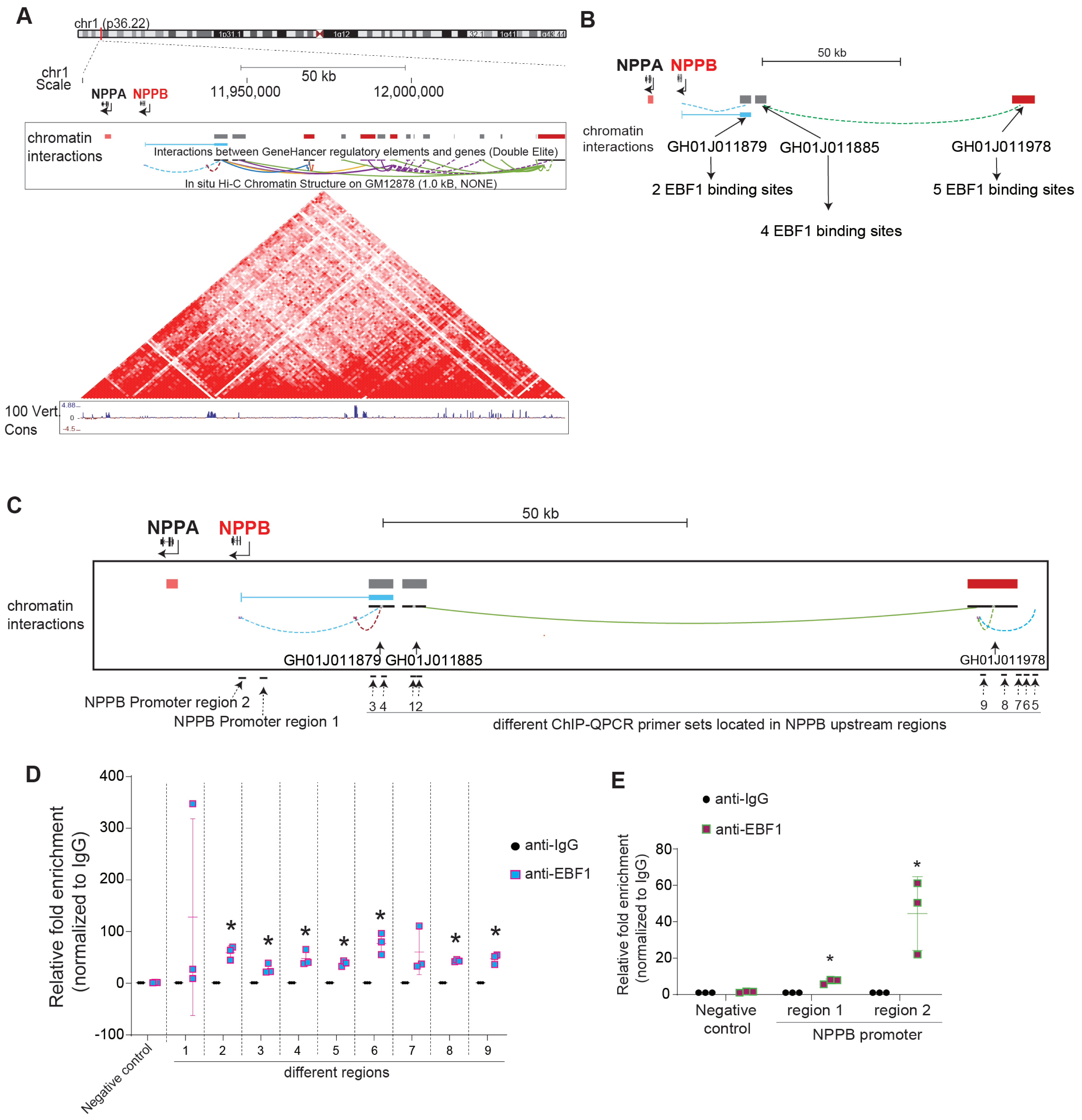

# Supplemental Figure 5

**A**

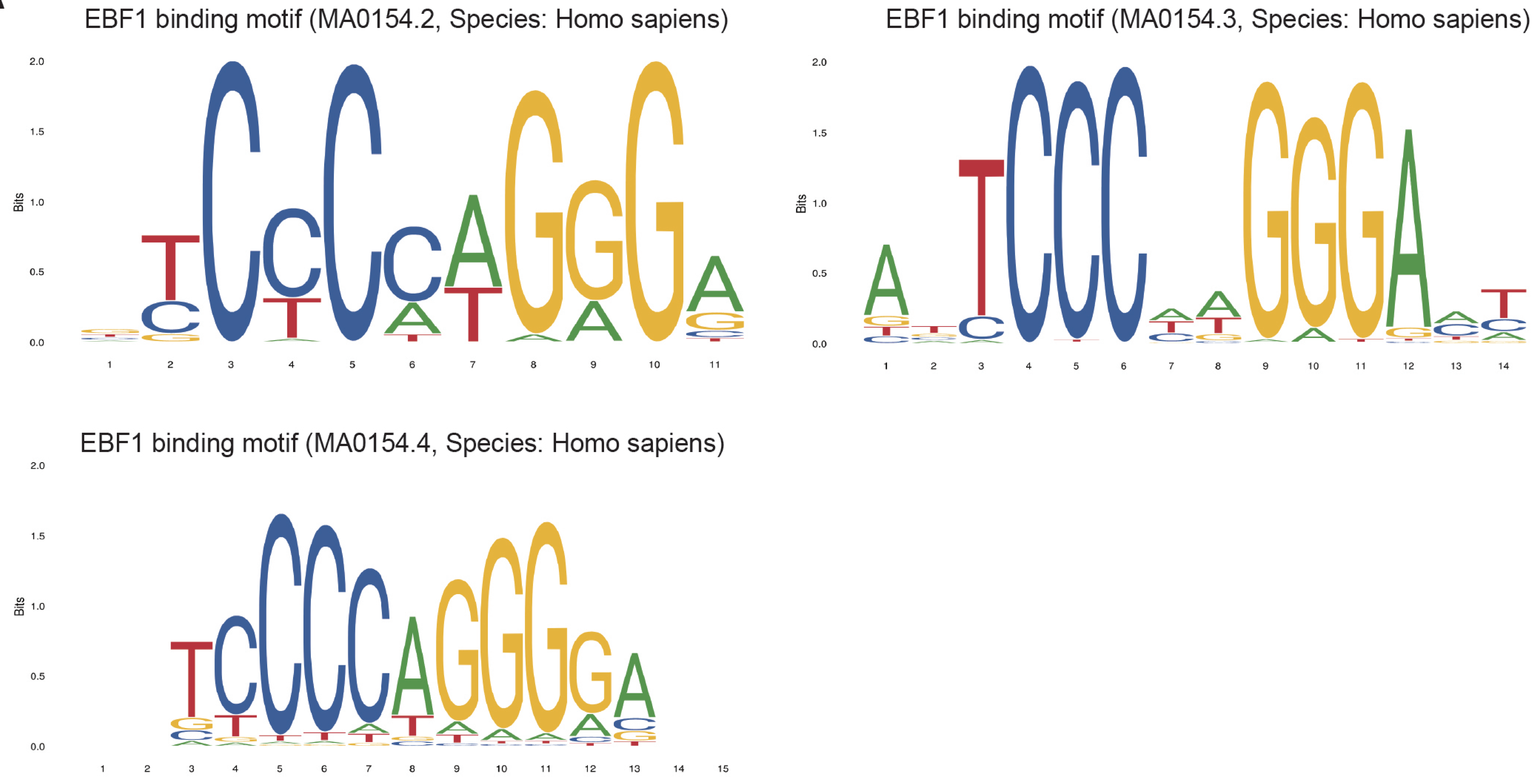


**B** **All EBF1 binding motifs Frequency matrix, Species: Homo sapiens**

>MA0154.2 EBF1

| | | | | | | | | | | | |
|---|---|---|---|---|---|---|---|---|---|---|---|
| A [ | 4513 | 0 | 0 | 886 | 0 | 9191 | 21366 | 1085 | 8427 | 0 | 22124 ] |
| C [ | 7097 | 10267 | 33855 | 22776 | 33781 | 22317 | 0 | 0 | 0 | 0 | 2891 ] |
| G [ | 12581 | 2643 | 0 | 0 | 0 | 0 | 0 | 32770 | 25294 | 33855 | 7215 ] |
| T [ | 9664 | 20945 | 0 | 10193 | 74 | 2347 | 12489 | 0 | 134 | 0 | 1625 ] |

>MA0154.3 EBF1

| | | | | | | | | | | | | | | |
|---|---|---|---|---|---|---|---|---|---|---|---|---|---|---|
| A [ | 1110 | 257 | 37 | 0 | 3 | 0 | 562 | 777 | 21 | 107 | 6 | 1544 | 604 | 250 ] |
| C [ | 115 | 276 | 232 | 1544 | 1544 | 1544 | 391 | 77 | 6 | 8 | 0 | 17 | 586 | 364 ] |
| G [ | 166 | 334 | 0 | 3 | 4 | 0 | 63 | 208 | 1544 | 1544 | 1544 | 84 | 145 | 81 ] |
| T [ | 153 | 678 | 1544 | 1 | 18 | 6 | 529 | 482 | 0 | 0 | 22 | 24 | 209 | 849 ] |

>MA0154.4 EBF1

| | | | | | | | | | | | | | | | |
|---|---|---|---|---|---|---|---|---|---|---|---|---|---|---|---|
| A [ | 14160 | 11403 | 2352 | 1321 | 568 | 648 | 2539 | 34745 | 3502 | 2617 | 1515 | 8856 | 31636 | 11229 | 11903 ] |
| C [ | 8868 | 9554 | 4506 | 34665 | 43256 | 42562 | 39675 | 1707 | 814 | 495 | 455 | 2462 | 6355 | 13299 | 10836 ] |
| G [ | 10891 | 13481 | 5497 | 1920 | 371 | 443 | 862 | 1723 | 38728 | 41651 | 42789 | 32761 | 4957 | 9855 | 9173 ] |
| T [ | 11506 | 10987 | 33070 | 7519 | 1230 | 1772 | 2349 | 7250 | 2381 | 662 | 666 | 1346 | 2477 | 11042 | 13513 ] |

**C**

**NKX2-5 Motif (p-value:1e-30)**

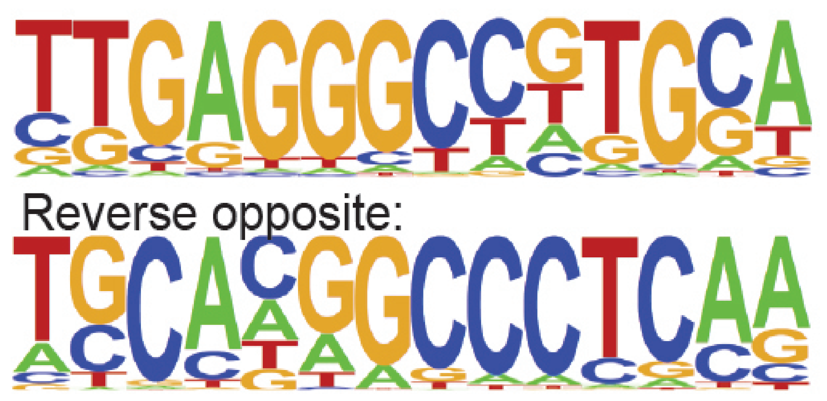


**D**

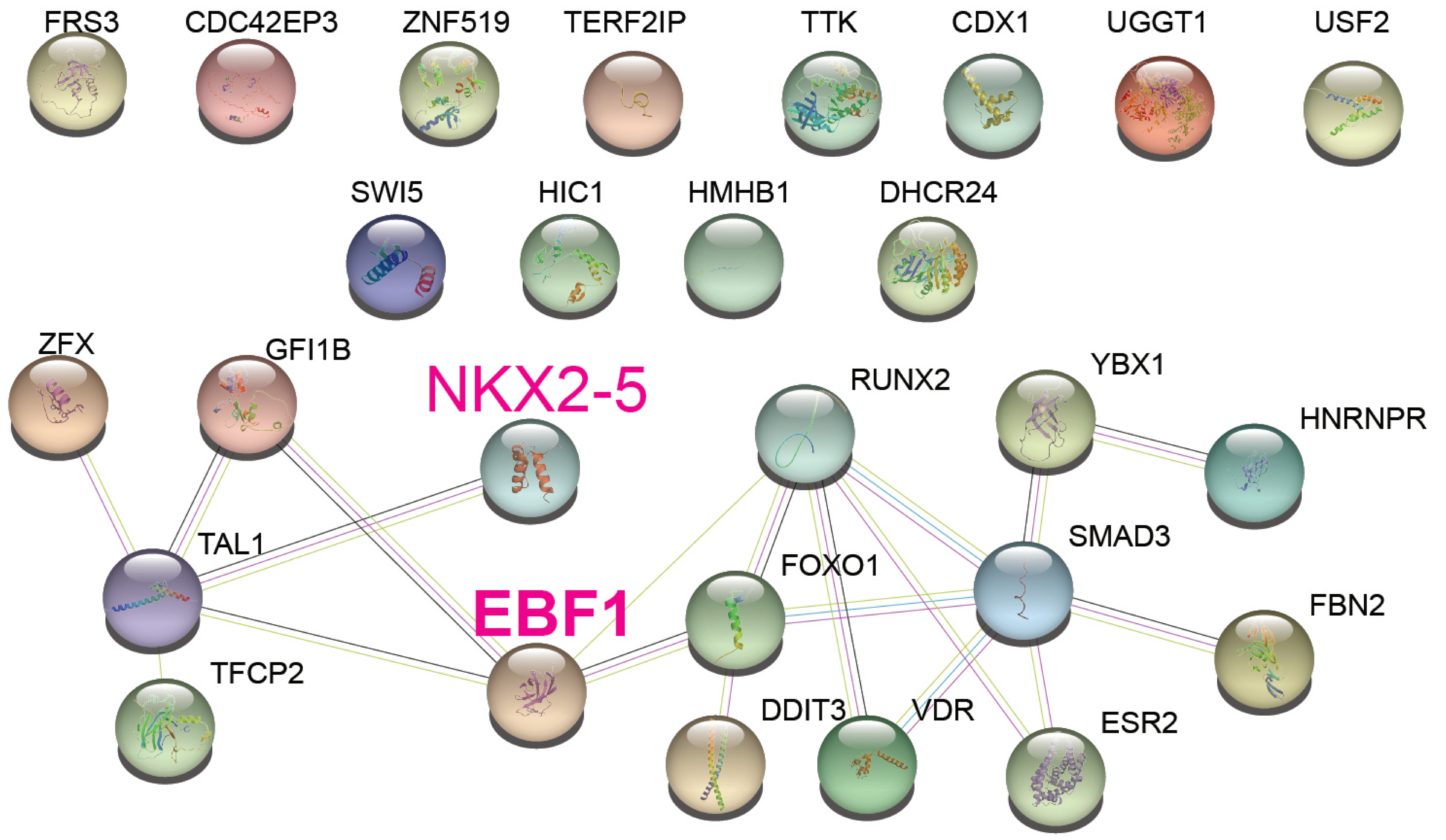

## Supplemental Figure 6

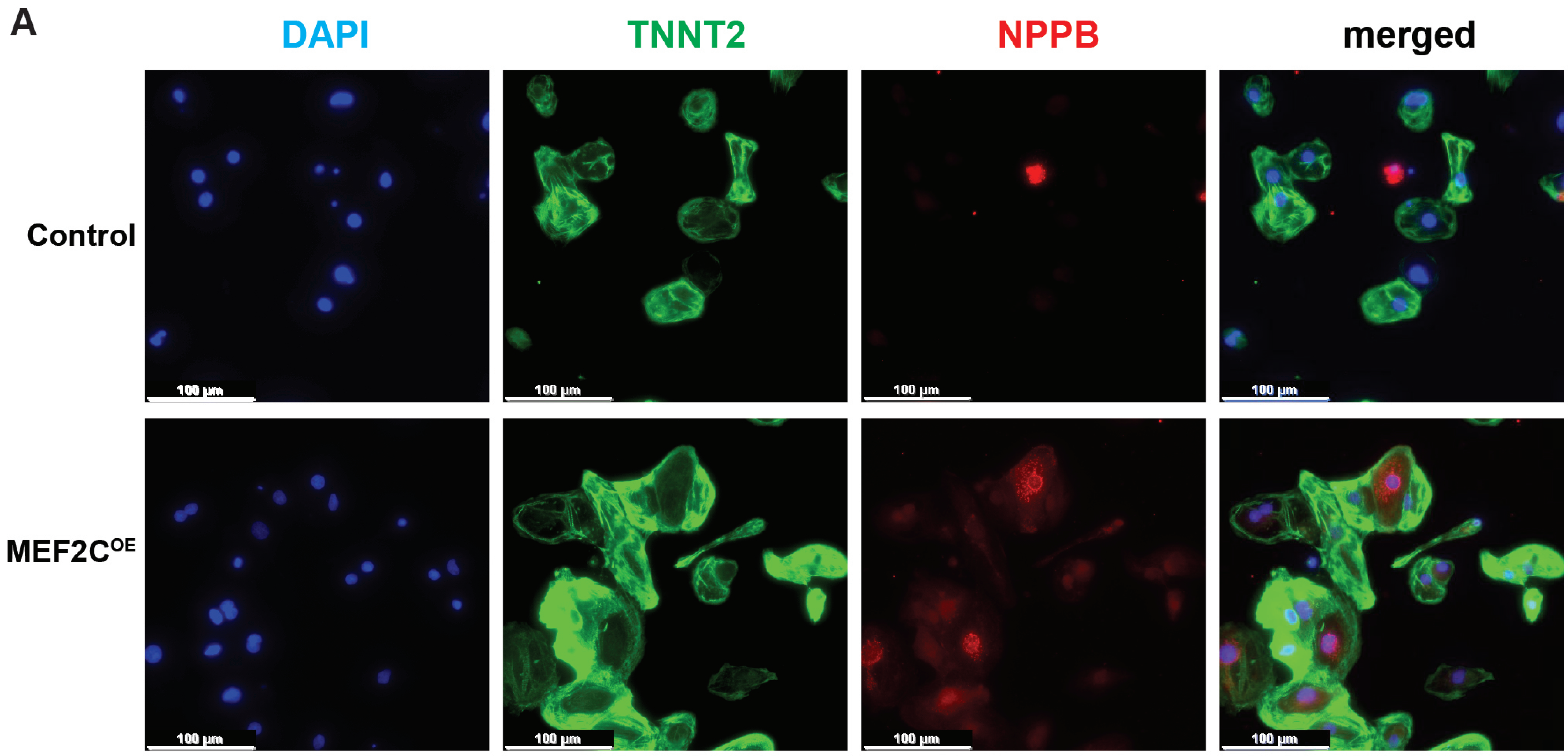


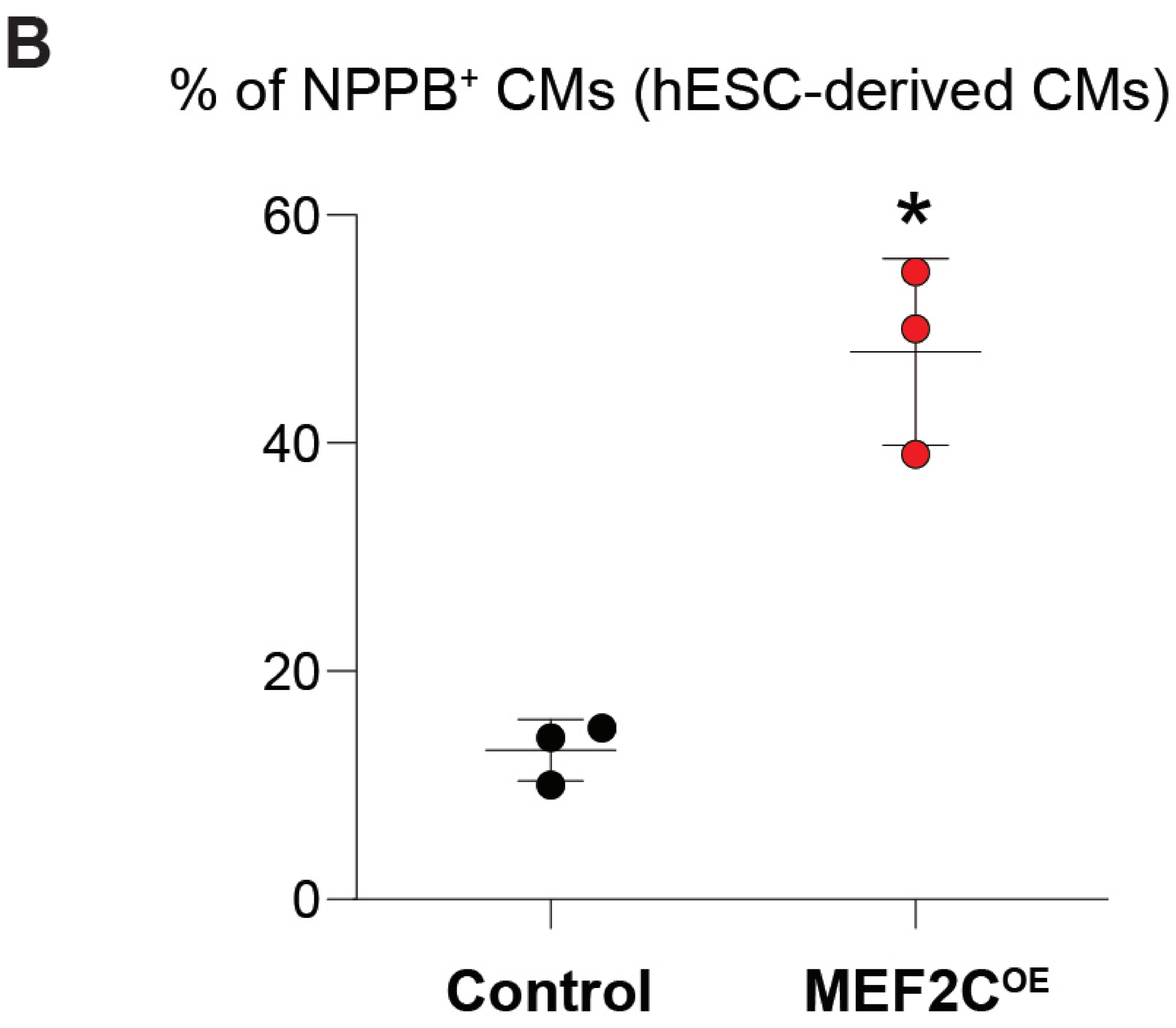

Supplement: Supplementary file 1 — Supplementary figures. [file thnov14p4894s1.pdf]
