# Supplementary material for: Transcriptome analysis reveals EBF1 ablation-induced injuries in cardiac system
Source: Theranostics. 2024 Aug 12;14(12):4894–915. doi: 10.7150/thno.92060 (PMC11373621; doi:10.7150/thno.92060)
Supplement: Supplementary file 2 — Supplementary tables. [file thnov14p4894s2.zip › Supplementary materials/Supplementary information.docx]

**KEY RESOURCES TABLE**

| **REAGENT or RESOURCE** | **SOURCE** | **IDENTIFIER** |
| --- | --- | --- |
| **Antibodies** | | |
| Mouse IgG1 Isotype Control | R&D system | Cat# MAB002; RRID:AB_357344 |
| Mouse Normal IgG Control antibody | Millipore | Cat# 12-371, RRID:AB_145840 |
| Rabbit Normal IgG Control antibody | Millipore | Cat# 12-370, RRID:AB_145841 |
| Normal Rabbit IgG antibody | Cell Signaling Technology | Cat# 2729, RRID:AB_1031062 |
| Cardiac Troponin T Ab | Thermo Fisher | Cat# MS-295-P, RRID:AB_61806 |
| APC Goat Anti-Mouse Ig (Multiple Adsorption)  Clone Polyclonal (RUO) | BD Biosciences | Cat# 550826, RRID:AB_398465 |
| Brachyury (D2Z3J) Rabbit mAb (TBXT antibody) | Cell Signaling Technology | Cat# 81694, RRID:AB_2799983 |
| Goat anti-Rabbit IgG (H+L) Cross-Adsorbed Secondary Antibody, Alexa Fluor 488 | Thermo Fisher | Cat# A-11008, RRID:AB_143165 |
| EBF-1 Antibody | Novus Biologicals | Cat# H00001879-D01P, RRID:AB_2097405 |
| NKX2.5 (E1Y8H) Rabbit mAb #8792 | Cell Signaling Technology | Cat# 8792, RRID:AB_2797667 |
| CD31 (PECAM-1) (D8V9E) XP® Rabbit mAb | Cell Signaling Technology | Cat #77699, RRID:AB_2722705 |
| α-Smooth Muscle Actin (D4K9N) XP® Rabbit mAb | Cell Signaling Technology | Cat #19245, RRID:AB_2734735 |
| Recombinant Anti-TCF21 antibody [EPR13449] (ab182134) | Abam | Cat #ab182134, RRID:AB_2889038 |
| Wheat germ agglutinin (WGA), fluorescein conjugate, Alexa Fluor™ 488 | Thermo Fisher | Cat# W11261, RRID: no data. |
| Mouse Anti-BrdU Monoclonal Antibody, Alexa Fluor 647 Conjugated, Clone 3D4 | BD Biosciences | Cat# 560209, RRID:AB_1645615 |
| BNP Polyclonal Antibody | Thermos Fisher | Cat: PA5-96084,  RRID: AB_2807886 |
| GAPDH (14C10) Rabbit mAb | Cell Signaling Technology | Cat: 2118,  RRID: AB_561053 |
| Anti-beta Actin antibody | Abcam | Cat: ab8227, RRID: AB_2305186 |
| COL1A1 Polyclonal Antibody | Thermos Fisher | Cat: PA5-29569,  RRID: AB_2547045 |
| Mouse IgG1 Isotype Control | R&D system | Cat# MAB002; RRID:AB_357344 |
| Mouse Normal IgG Control antibody | Millipore | Cat# 12-371, RRID:AB_145840 |
| Rabbit Normal IgG Control antibody | Millipore | Cat# 12-370, RRID:AB_145841 |
| Normal Rabbit IgG antibody | Cell Signaling Technology | Cat# 2729, RRID:AB_1031062 |
| Cardiac Troponin T Ab | Thermo Fisher | Cat# MS-295-P, RRID:AB_61806 |
| APC Goat Anti-Mouse Ig (Multiple Adsorption)  Clone Polyclonal (RUO) | BD Biosciences | Cat# 550826, RRID:AB_398465 |
| Brachyury (D2Z3J) Rabbit mAb (TBXT antibody) | Cell Signaling Technology | Cat# 81694, RRID:AB_2799983 |
| **Chemicals, Peptides, and Recombinant Proteins** | | |
| Saponin | Sigma | S4521-25G |
| BsmBI | NEB | R0580L |
| BbsI | NEB | R0539L |
| 16% Paraformaldehyde (formaldehyde) aqueous solution (for flow cytometry and Immunofluorescence) | ELECTRON MICROSCOPY SCIENCES | 15710 |
| Puromycin dihydrochloride | Sigma-Aldrich | P8833-10MG |
| Polybrene | Sigma-Aldrich | H9268-5G |
| TrypLE™ Express Enzyme (1X), phenol red | Gibco | 12605010 |
| DAPI Fluoromount-G | southernbiotech | 0100-20 |
| **Critical Commercial Assays** | | |
| EZ-Magna ChIP™ A/G Chromatin Immunoprecipitation kit | Millipore | 17-10086 |
| DMEM/F12 | Gibco | 11320082 |
| Corning® Matrigel® Growth Factor Reduced (GFR) Basement Membrane Matrix, *LDEV-Free, 10mL | Corning | 354230 |
| CellMaxx™ Bovine Albumin, Low Free Fatty Acid, Stem Cell Culture | Fisher scientific | 199899 |
| truChIP™ Chromatin Shearing Kit | Covaris | PN 520127 |
| Surveyor® Mutation Detection Kit for Standard Gel Electrophoresis | Integrated DNA Technologies, Inc. | 706021 |
| DNeasy Blood & Tissue Kit (50) | QIAGEN | Cat No./ID: 69504 |
| RNeasy Mini Kit | QIAGEN | 74104 |
| High-Capacity RNA-to-cDNA™ Kit | Applied Biosystems | 4387406 |
| DreamTaq Green PCR Master Mix (2X) | Thermo Scientific | K1081 |
| Fast SYBR Green Master Mix | Applied Biosystems | 4385612 |
| Albumin, Bovine Serum, Fraction V, Low Heavy Metals | Sigma-Aldrich | 12659 |
| Pierce™ ECL Western Blotting Substrate | Pierce | 32209 |
| Pierce™ Classic Magnetic IP/Co-IP Kit | Pierce | 88804 |
| cOmplete™ Lysis-M EDTA-free | Roche | 04719964001 |
| **Experimental Models: Cell Lines** | | |
| HEK 293T cells | ATCC | CRL-3216 |
| Wild type (WT) Human ESC line H9 | ATCC | HTB176 |
| Wild type (WT) Human iPSCs | This paper | N/A |
| **Oligonucleotides** | | |
| Primers see Table S1. Oligonucleotides | This paper | N/A |
| **Recombinant DNA** | | |
| psPAX2 | From Dr. Gang Hu lab (NIH) | N/A |
| pMD2.G | From Dr. Gang Hu lab (NIH) | N/A |
| lentiCRISPRv2-puro vector | Addgene | N/A |
| **Software and Algorithms** | | |
| Image J | National Institutes of Health | https://imagej.nih.gov/ij/ |
| FlowJo (Treestar) | FlowJo, LLC | https://www.flowjo.com/about/company |
| Bowtie2 |  | Langmead and Salzberg, 2012 |
| MACS2 |  | Zhang et al., 2008 |
| edgeR |  | (McCarthy et al., 2012; Robinson et al., 2010 |
| Adobe Illustrator | Adobe Inc. | https://www.adobe.com/products/illustrator.html |
| Metacore | Clarivate Analytics | https://portal.genego.com/ |
| DNASE2TF |  | Sung et al., 2014 |
| **Bacterial and viral strains** | | |
| MAX Efficiency™ DH5α Competent Cells | Invitrogen | 18258012 |
| One Shot™ Stbl3™ Chemically Competent E. coli | Invitrogen | C737303 |
|  |  |  |
